# Combining structural and bioactivity-based fingerprints improves prediction performance and scaffold hopping capability

**DOI:** 10.1186/s13321-019-0376-1

**Published:** 2019-08-08

**Authors:** Oliver Laufkötter, Noé Sturm, Jürgen Bajorath, Hongming Chen, Ola Engkvist

**Affiliations:** 1 Hit Discovery, Discovery Sciences, R&D, AstraZeneca, Gothenburg, Sweden; 2 Department of Life Science Informatics, B-IT, LIMES Program Unit Chemical Biology and Medicinal Chemistry, Rheinische Friedrich-Wilhelms-Universität, Bonn, Germany

**Keywords:** Machine learning, Random forest, High throughput screening, Activity prediction, HTSFP, ECFP, Circular fingerprints, Scaffold hopping

## Abstract

This study aims at improving upon existing activity predictions methods by augmenting chemical structure fingerprints with bio-activity based fingerprints derived from high-throughput screening (HTS) data (HTSFPs) and thereby showcasing the benefits of combining different descriptor types. This type of descriptor would be applied in an iterative screening scenario for more targeted compound set selection. The HTSFPs were generated from HTS data obtained from PubChem and combined with an ECFP4 structural fingerprint. The bioactivity-structure hybrid (BaSH) fingerprint was benchmarked against the individual ECFP4 and HTSFP fingerprints. Their performance was evaluated via retrospective analysis of a subset of the PubChem HTS data. Results showed that the BaSH fingerprint has improved predictive performance as well as scaffold hopping capability. The BaSH fingerprint identified unique compounds compared to both the ECFP4 and the HTSFP fingerprint indicating synergistic effects between the two fingerprints. A feature importance analysis showed that a small subset of the HTSFP features contribute most to the overall performance of the BaSH fingerprint. This hybrid approach allows for activity prediction of compounds with only sparse HTSFPs due to the supporting effect from the structural fingerprint.

**Electronic supplementary material:**

The online version of this article (10.1186/s13321-019-0376-1) contains supplementary material, which is available to authorized users.

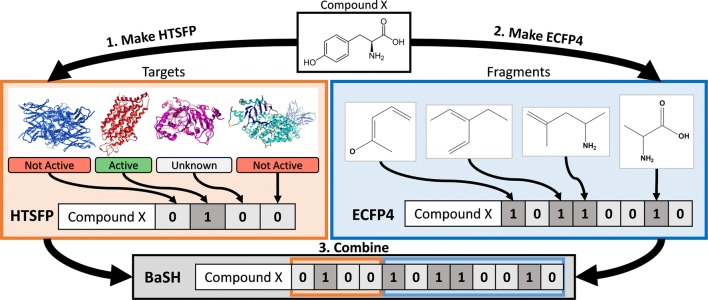

## Introduction

The traditional and most intuitive method of predicting compound activity is through the use of structure activity relationship (SAR) models. Logically, compounds with similar structural features or scaffolds would express similar activities. While SAR-based activity predictions are a practical and often effective method, the predictions made are based on structural similarity and therefore are inherently limited in structural diversity. This limits the scaffold hopping potential or exploration of chemical space and impedes the identification of novel active compounds. Another limitation of structure based fingerprints is the existence of activity cliffs, this is where two compounds with high degrees of similarity express inverse activity relationships towards a target. It is therefore very difficult to distinguish such compounds using structural descriptors. To bypass the drawbacks of SAR models, historical bioactivity data can be used to build fingerprints for each compound which can subsequently be applied in machine learning to make compound property predictions independent of chemical structural information. Such predictive models have been built using bioactivity data obtained from various sources, e.g. transcriptomics [1], cell imaging [2], affinity/inhibition data [3, 4], or high throughput screening (HTS) [5–7]. Such bioactivity data has been utilized for a number of applications such as compound safety/toxicity predictions [1, 8], compound potency/activity predictions [3–5, 9, 10], target elucidation [11], or elucidation of compound MoA [12, 13]. A review by Wassermann et al. provides an in-depth summary of the history and applications of historical bioactivity data to date [14]. A study by Kauvar et al. presented one of the earliest applications of bioactivity profiles for compound property prediction [3]. In this study, affinity profiles of compounds based on a panel of 18 olfactory proteins were generated and used to predict compound binding properties on external targets. A study presented by Fliri et al. used a somewhat larger database to build bioactivity profiles termed ‘biospectra’ to predict compound-target activities [4]. This bioactivity profile was based on a panel of 1567 compounds and 92 assays representing a diverse cross-section of the proteome.

HTS is a method used for large scale testing of compound libraries, containing up to five million compounds, against a single target [15]. HTS has become feasible during the last three decades due to advances in process automation along with the development of new technologies [15, 16]. HTS is a resource-intensive process, which usually only identifies a very small portion of active compounds [17]. To reduce resource costs in HTS, compound activity prediction methods can be employed. Using machine learning together with either structural or bioactivity descriptors, predictive models can be built. The limiting factor in using bioactivity based methods is the lack of data, meaning this method can only be applied to existing compounds which have sufficient bioactivity data. Structural descriptors can be useful for predicting a variety of compound properties [18]. Structure based descriptors such as ECFP/Morgan circular fingerprints are an effective and established method for predicting compound activity [6, 19, 20], although the structural diversity of predictions can be limited by the training data. To overcome this potential drawback Petrone et al. introduced a bioactivity based descriptor derived from historical HTS data i.e. the HTS Fingerprint (HTSFP) [5]. The HTSFP has the advantage of not containing any structural information and thereby can be used to make activity predictions independent of any structural features. Moreover, in phenotypic screens HTS fingerprints may detect active compounds with distinct MoAs, such as alternate binding sites. Unfortunately, the HTSFP has one major drawback, which is that predictions cannot be made for all compounds but only for compounds that have been previously tested in HTS assays, compounds without any HTS data cannot have an HTSFP. Furthermore, compounds with very sparse HTSFPs i.e. compounds having only been tested in very few assays, have limited practicality in such predictive models. These compounds are often not useful as they introduce noise into the data and reduce the predictive performance of models and therefore are removed from the dataset. A fingerprint density cutoff is commonly used to exclude these compounds [21, 22]. This method of data processing leads to the loss of a significant amount of potentially valuable information. Despite these problems HTSFPs have proven to be an effective and robust tool for compound activity predictions in a number of retrospective studies.

Petrone et al. compared the performance of HTSFP and ECFP4 and showed that the HTSFP had better performance for certain targets. The most prominent aspect of this study was the increased structural diversity of the HTSFP predictions [5]. Paricharak et al. showed that HTSFPs are effective tools for iterative screening approaches in HTS to provide more targeted and efficient screening, saving costs and resources [10]. More recently, HTSFPs have been employed for multitask machine learning methods. The study by Sturm et al. compared HTSFP and ECFP4, again showing that the predictions returned from HTSFP models have little overlap with those of the ECFP, concluding that HTSFPs are valuable tools for scaffold hopping [20, 22]. A study by Wassermann et al. in 2013 showed the first step in the direction of combining structural and bioactivity descriptors [9]. Their study focused on generating HTSFPs for compounds which had no available HTS data. This was performed by calculating an untested compounds structural similarity to compounds with existing HTSFPs. The HTSFP of compounds with high similarity were substituted onto the untested compounds. A different study by Riniker et al. went a step further and described a method of using both ECFP4 and HTSFPs for activity prediction by building machine learning models on each of the two descriptor types individually and subsequently combining the two trained models using heterogeneous classifier fusion for the final activity predictions [6].

In this study, a novel fingerprint was designed by combining bioactivity descriptors (HTSFPs) with structural descriptors. The aim was to improve compound activity predictions and scaffold hopping potential of structural fingerprints while also showing that the method of combining different types of descriptors can in general be beneficial in terms of synergistic effects. This method is developed with the prospect of improving iterative screening approaches, through targeted compound set selection with greater accuracy and coverage of chemical space. The underlying idea was that combining the fingerprints fortifies the HTSFP with structural data, thereby removing the necessity of having to make a HTSFP density cutoff and allowing for a more efficient use of available HTS data. The fingerprint introduced herein was designed by concatenating the HTSFP with an ECFP4 to make a bioactivity-structure hybrid (BaSH) fingerprint. The HTSFP was constructed using HTS data from PubChem made up of 561 assays and is based on the activity flags set in the PubChem database. A random forest binary classifier was used to build the predictive model. The results were validated via a retrospective analysis on a set of HTS assays which had been excluded from the training data, i.e. these assays were not included in the HTSFP or BaSH fingerprint. The results were benchmarked against the individual HTSFP and ECFP4.

## Results and discussion

The HTS data was obtained from PubChem bioassays and post-refinement contained a total of 715,000 unique compounds and 561 HTS assays. A retrospective analysis was performed using separate test and validation sets. A set of ten test assays were randomly chosen and excluded from the HTSFP and BaSH fingerprint and used for the hyperparameter optimization. Another 24 assays where chosen at random from the HTS dataset as the validation set and were excluded from the HTSFP and BaSH fingerprint, a detailed overview of these assays is shown in Table 1. These 24 validation set assays did in some cases have a biological overlap with the assays in the HTSFP. While this overlap was not investigated prior to building the predictive models, the overlap is discussed for the relevant assays further on in the results. The results from the bioactivity-structure hybrid (BaSH) fingerprint were benchmarked against the un-concatenated HTSFP and ECFP4. Furthermore, the scaffold hopping potential of the BaSH fingerprint was investigated by comparing topological scaffolds and performing a nearest neighbor comparison. The random forest classifier models built on the ECFP4, HTSFP, and BaSH fingerprint were used to make predictions for each assay. The results of the random forest analysis were investigated for each of the three fingerprint types using a variety of different performance metrics most of which are derived from values of the confusion matrix. Each metric was averaged using the results of a sixfold cross validation and are discussed in detail in the following paragraphs. An overview of all metrics and the confusion matrix for all assays can be found in Additional file 1: Table S2.

**Table 1:** Overview of the 24 test assays used in the validation set

AID	Compounds tested	Actives	% Actives	Target information	Assay type
522	64907	1225	1.89%	Nuclear receptor Steroidogenic Factor 1 (SF-1)	Cell-based
527	24074	64	0.27%	Bacterial Quorum Sensing	Cell-based
555	65239	316	0.48%	Mevalonate kinase	Biochemical
560	64907	979	1.51%	Retinoic Acid Receptor-related orphan receptor A (RORA)	Cell-based
746	59787	366	0.61%	c-Jun N-Terminal Kinase 3 (JNK3)	Biochemical
798	218716	302	0.14%	Coagulation factor XIa	Biochemical
1006	195564	2976	1.52%	Compounds inhibiting luciferase	Biochemical
1273	127297	1153	0.91%	Insulin promoter activity—Proinsulin	Cell-based^a^
1515	217964	445	0.20%	Retinoblastoma binding protein 9 (RBBP9)	Biochemical
2129	315002	2199	0.70%	BCL2-related protein, long isoform (BCLXL).	Biochemical
2280	324750	1419	0.44%	GLD-1 protein—TGE RNA interaction.	Biochemical
2540	330397	4119	1.25%	Sentrin-specific protease 8 (SENP8)	Biochemical
2544	330397	393	0.12%	Intestinal alkaline phosphatase	Biochemical
2553	305614	3253	1.06%	Transient receptor potential cation channel C6 (TRPC6)	Cell-based^a^
2606	324751	157	0.05%	Membrane-associated serine protease Rv3671c	Biochemical
463104	331676	1100	0.33%	Adaptive arm of the Unfolded Protein response	Cell-based
504406	323914	194	0.06%	UDP-galactopyranose mutase (UGM) enzyme	Biochemical
504454	339285	1446	0.43%	Beta-2AR agonists-b2AR	Cell-based
588497	340322	780	0.23%	Botulinum neurotoxin light chain F protease	Biochemical
602363	347157	446	0.13%	Modulators of the fidelity of start codon recognition	Cell-based
623901	332759	470	0.14%	Inhibitors of miR-122 (miRNA)	Cell-based
624414	400339	482	0.12%	Mucolipin-1 Transient Receptor Potential 1 (TRPML1)	Cell-based
686964	369939	1149	0.31%	Methyl-CpG binding domain protein 2	Biochemical
720700	369939	3123	0.84%	Phospholipase C, gamma 1	Biochemical

Shown are their PubChem AID, total number of compounds tested in assay, and the proportion of active compounds, assay target information, and assay type. Compounds are labeled active or inactive based on the activity flag set in the PubChem data

^a^Assay types were not indicated in PubChem for these assays and were interpreted manually

### Classification performance

#### Receiver operator characteristic

**Fig. 1:**
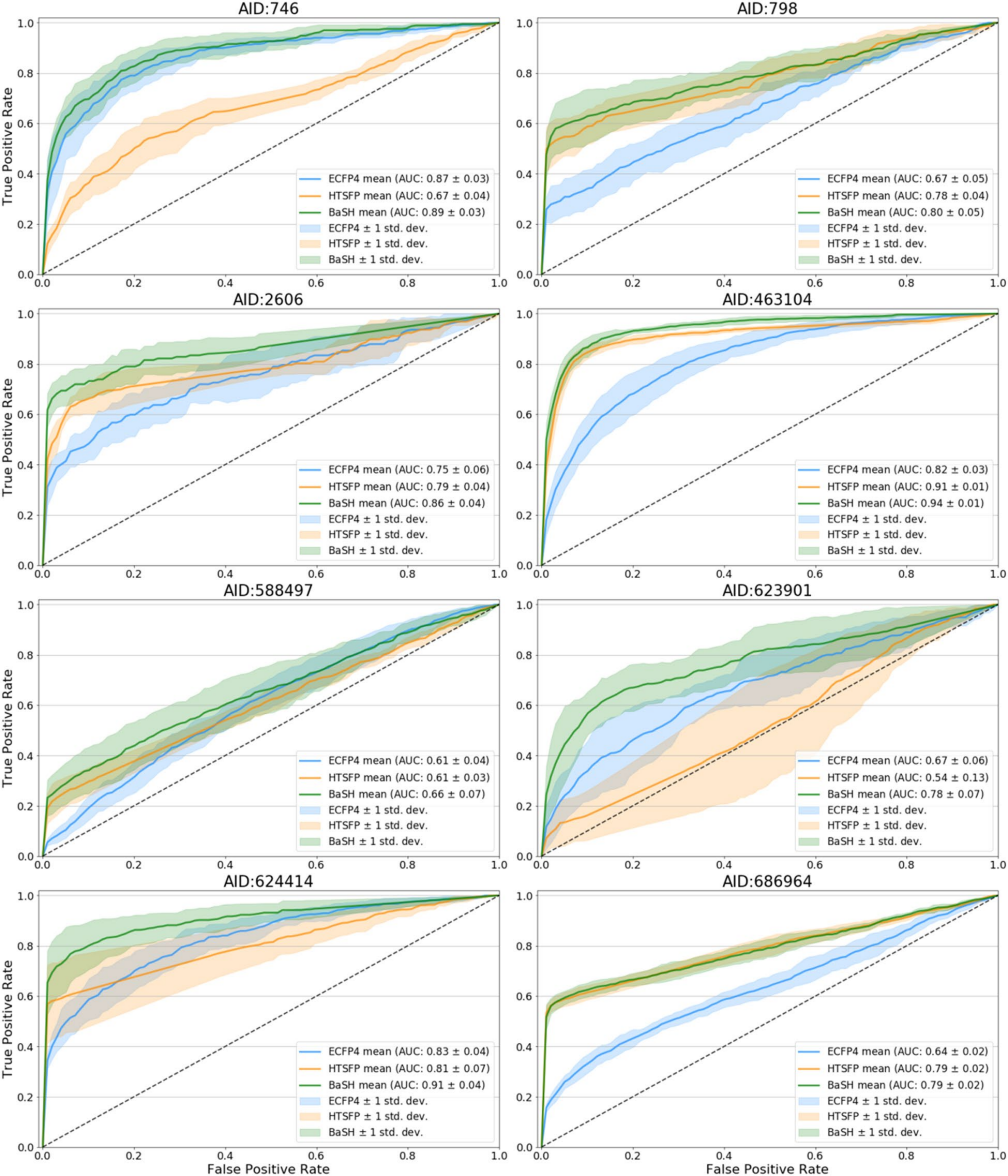
Receiver operator characteristic (ROC) curves comparing the hybrid fingerprint (BaSH) with the HTSFP and ECFP4, green, orange, and blue respectively. The shaded area either side of the ROC curve represents one standard deviation. Shown are 8 of the 24 validation set assays with the most diverse results

**Fig. 2:**
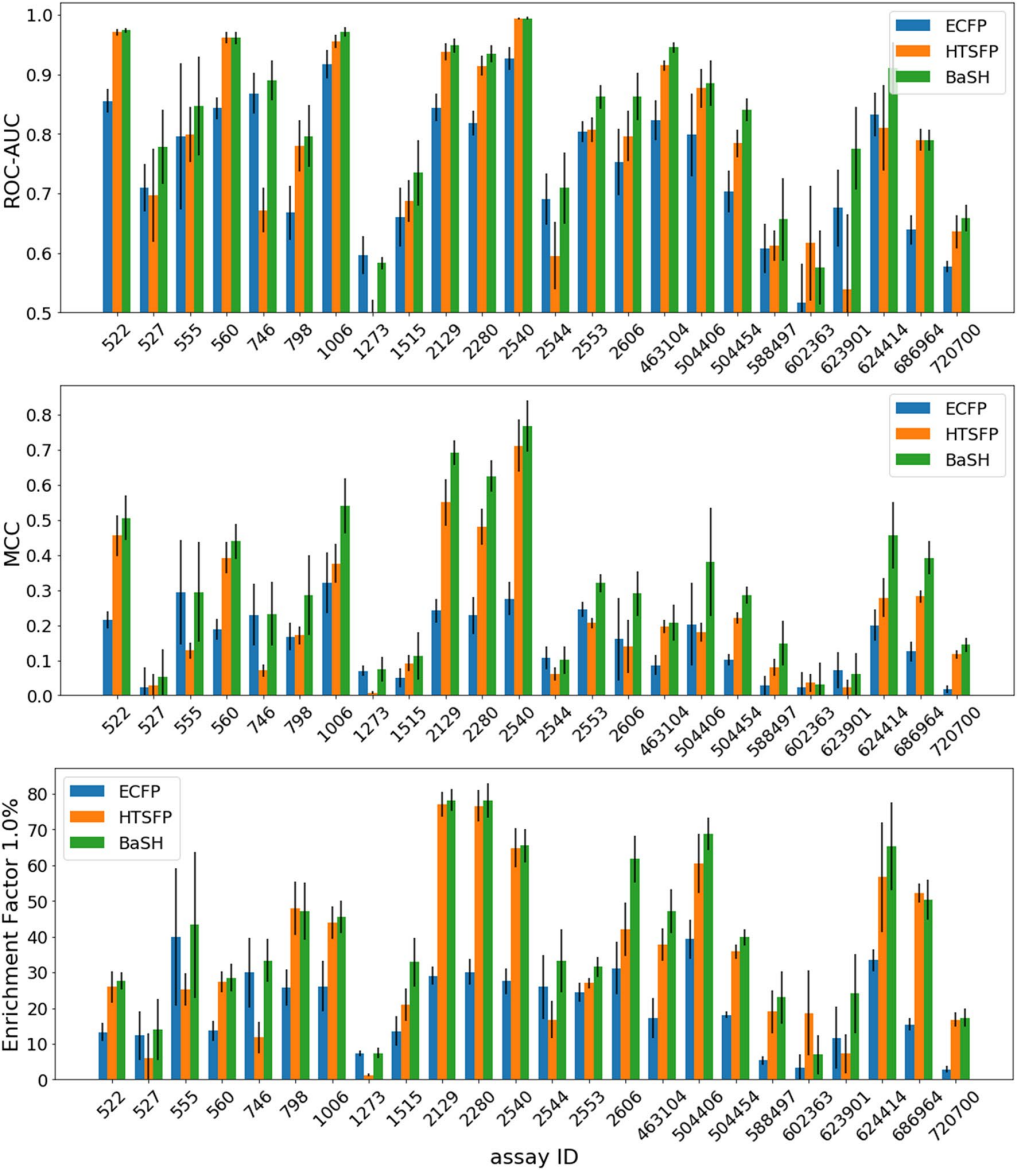
Performance metrics for the 24 test assays comparing the hybrid fingerprint (BaSH) with the HTSFP and the ECFP4, green, orange, and blue respectively. Top: ROC-AUC, Middle: Matthews correlation coefficient, Bottom: enrichment factor. The errors bars in black represent one standard deviation

Receiver-operator-characteristic (ROC) curves for each of the three fingerprint types and eight of the 24 test assays are displayed in Fig. 1. The ROC area-under-curve (ROC-AUC), shown in Fig. 2 bar plot, were calculated to compare the relative performances between the three fingerprint models. The ROC curve compares the true positive rate (TPR) against the false positive rate (FPR), while varying the threshold of the classification confidence scores, this provides an indication of the early enrichment and gives a rough idea of the overall performance. Analysis of these curves and AUC values indicates that prediction performance of the ECFP was better than the HTSFP in only seven of the 24 test assays. The original study on HTSFPs by Petrone et al. showed that the ECFP was a more reliable descriptor than HTSFP in terms of ROC AUC [5]. The HTSFP used in Petrone’s study was based on 195 assays which may have limited its potential performance compared with 651 assays used in this study. Other recent studies also show that the HTSFP often outperforms the ECFP in terms of ROC AUC, but credit this in part to the presence of confirmatory or similar assays [6, 20]. The relative performance between the ECFP and HTSFP varied from assay to assay, which is likely dependent on the assay target types and also on the density of the HTSFPs for the compounds tested in each assay. Some of the test assay targets have also been tested in other assays or have closely related targets in other assays, thereby boosting the predictive performance of these particular assays. The BaSH fingerprint predictions showed increased ROC for 18 of the 24 test assays, although in the remaining seven assays the BaSH fingerprint showed comparable performance to the better of the HTSFP and ECFP. Noticeably the ROC curves showed that the early enrichment appeared to be improved in most test assays.

#### Precision, recall, and F1 score

The precision, recall and F1 scores were calculated for each of fingerprints, these were based on the averages from the sixfold cross validation and are plotted in Additional file 1: Figure S3. The HTSFP had relatively low precision compared to the ECFP, for all but five of the 24 test assays but inversely had the highest recall/sensitivity in every test assay except one (623901). The BaSH fingerprint performed best in all 24 assays for precision but was out performed by the HTSFP in the sensitivity scores in all but one assay (623901), although the BaSH fingerprint sensitivity was still better than that of the ECFP4 In the majority of cases. This shows that the HTSFP is predicting a very large number of compounds to be active, thereby recovering a large portion of the true positives, i.e. the high sensitivity, but at the same time a large number of false positives are predicted giving the HTSFP a low precision. To further analyze these results the F1 scores were compared, which gave the harmonic mean of the precision and sensitivity. The plot of the F1 scores resolves the previously unclear results, showing that the BaSH fingerprint is on par, or outperforming the other two fingerprints in all test assays. The trends seen in the F1 scores are similar to those of the MCC analysis seen in Fig. 2.

### Mathews correlation coefficient

Results were also compared using the Mathews correlation coefficient (MCC). This is a well-suited metric for measuring the predictive quality on very unbalanced datasets, which is the case for this data, having an average active compound rate of 0.80% across the 561 assays used. The average proportion of actives across the 24 tested assays was 0.57% which is very imbalanced and can affect the quality of other measurement techniques. The bar plot in Fig. 2 compares the average MCC scores from the sixfold cross validation for each of the three fingerprint types. In eight of the 24 test assays the ECFP4 outperformed the HTSFP by a clear margin, in fourteen assays the HTSFP scored higher than the ECFP4 and in the remaining two MCC scores were similar. Again, the varying relative performances between these two fingerprints is likely highly dependent on the amount of information in the assay compounds’ HTS fingerprints, therefore test assays who have biological overlap with the assays of the fingerprint will have better performance and also assays with large portions of mostly sparse HTSFPs will be expected to perform poorer. The MCC scores for the BaSH fingerprint are higher than those of the other two fingerprints for all but five of the 24 test assays.

#### Cohen’s Kappa score

The Cohen’s kappa scores were also calculated from the cross-validation results and are plotted in Additional file 1: Figure S3. The plot again shows the improved performance of the BaSH fingerprint compared with the other two fingerprints. The Kappa score shows an identical trend to that seen in the MCC plot.

#### Enrichment

To further investigate the relative performances of the three different fingerprints, the top scoring 1% of compounds from each prediction run of the cross validation were compared. The top 1% represented between 240 and 4000 compounds, depending on the assay (see Table 1). The enrichment factor (EF_1%_) for each assay was determined. The average enrichment factor for each fingerprint type in each assay is shown in Fig. 2. The ECFP4 showed the poorest enrichment in 18 of the 24 test assays. Overall, the BaSH fingerprint produced the on par or better enrichment factor for all test assays except one (assay 602363). In many cases the EF_1%_ differences were only marginal but the EF of the BaSH fingerprint did become slightly more apparent when taking into account a higher percentage of compounds, i.e. EF_1.5%_, EF_2%_, EF_2.5%_.

### Scaffold hopping analysis

#### Scaffold overlap

**Fig. 3:**
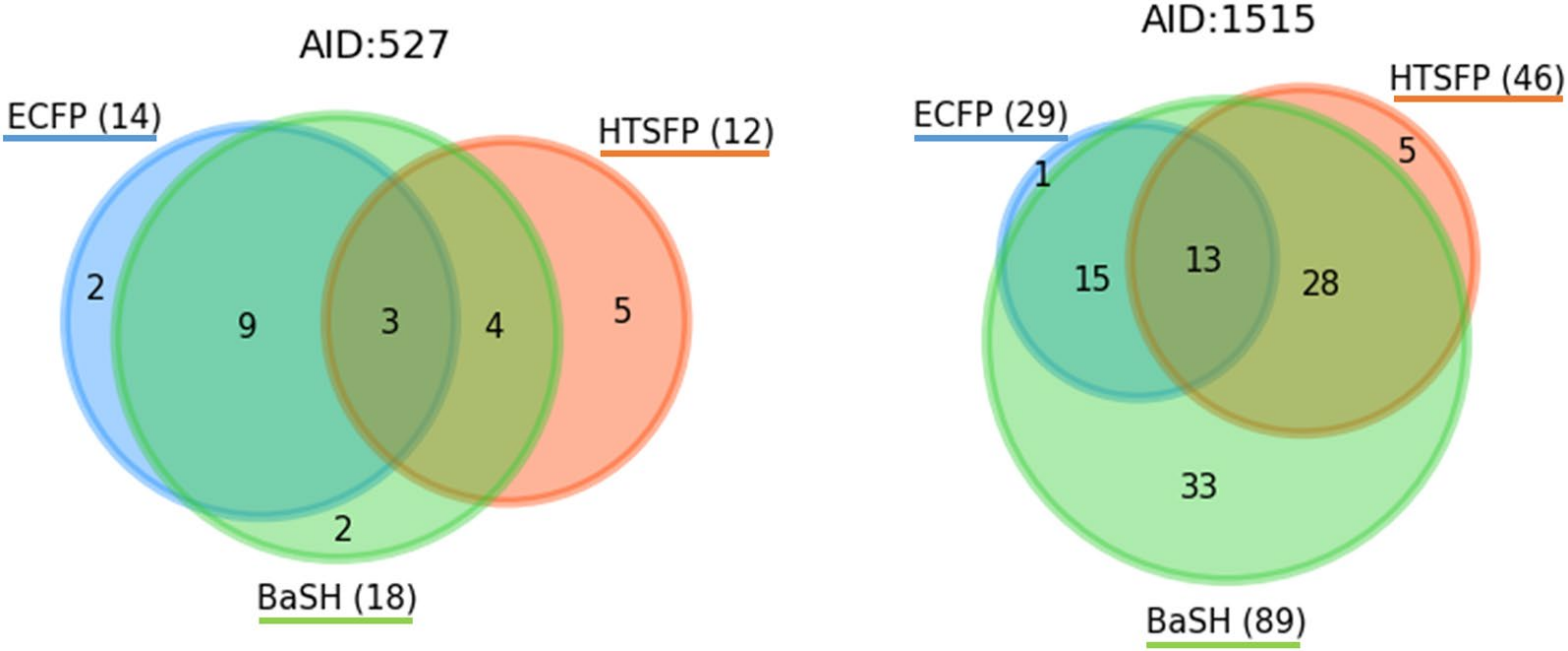
Venn diagrams showing the number of unique topological-Murcko scaffolds in the top scoring 1% of predictions. Each circle represents one of the three predictive models: BaSH, HTSFP, and ECFP4 (green, orange, blue respectively). Left diagram refers to test assay 527 and right diagram to test assay 1515

The second goal of the study was to determine the scaffold hopping potential of the BaSH fingerprint compared with the ECFP4 and the HTSFP. The scaffold hopping capabilities of the HTSFP is well known and has been demonstrated in a number of studies [5, 20]. To compare the chemical diversity of the predicted compounds, the topological-Murcko scaffolds of each of the true positive predicted compounds in the top scoring 1% of predictions were compared. The topological-Murcko scaffold is created by removing all side chains and subsequently converting all atoms in the structure to sp3 carbons. As expected the scaffolds predicted using the HTSFP had only a limited overlap with the scaffolds predicted using the ECFP4. On average, 59% of the scaffolds from the ECFP4 were also detected by HTSFP. Venn diagrams were constructed for the three fingerprint types and are shown in Fig. 3. The Venn diagrams in Fig. 3 show the total number of unique scaffolds detected by each descriptor next to the descriptor name. The blue orange and green circles represent the unique scaffolds retrieved by the ECFP, HTSFP, and BaSH respectively. The numbers in each segment of the circles correspond to the number of unique scaffolds found in that segment. The number of scaffolds is proportional to the sizes of the circles. Combining the structural (ECFP4) and bioactivity (HTSFP) fingerprints into one fingerprint (BaSH), one would expect the therefrom predicted scaffolds to reflect some form of overlap from the predictions of both the other two fingerprint types. Assays 527 and 1515 are representatives of the two extremes within the 24 test assays and are shown in Fig. 3. In the case of assay 1515, a very wide separation between the three scaffold groups can be seen, whereas in assay 527 the BaSH overlaps with almost all the scaffolds of both the ECFP4 and the HTSFP. The latter is the expected result, which shows no or very few novel scaffolds relative to the ECFP and HTSFP. This distribution pattern seen in the Venn diagram for assay 527 was not very common among the other 24 test assays. Interestingly, the BaSH fingerprint also predicted an additional completely unique set of topological scaffolds that did not overlap with either of the ECFP4 or the HTSFP predictions in all test assays (green shaded area). This effect was most pronounced in assay 1515 showing 37% unique scaffolds predicted only by the BaSH fingerprint. On average, the BaSH fingerprint predicted 16% unique scaffolds across the 24 test assays. The 33 scaffolds unique to assay 1515 (see Fig. 3) were investigated more closely, an example of six of these structurally diverse compounds predicted correctly only by the BaSH are shown in Additional file 1: Figure S8. These results indicate synergistic effects when combining the two fingerprints, leading to the detection of additional novel scaffolds. The overall count of true positive scaffolds predicted within the top scoring 1% of compounds was also highest for the BaSH fingerprint in most test assays. This suggested that the BaSH fingerprint was a more effective fingerprint for scaffold hopping than its precursors. Venn diagrams of all test assays can be found in the additional data Fig. 4.

**Fig. 4:**
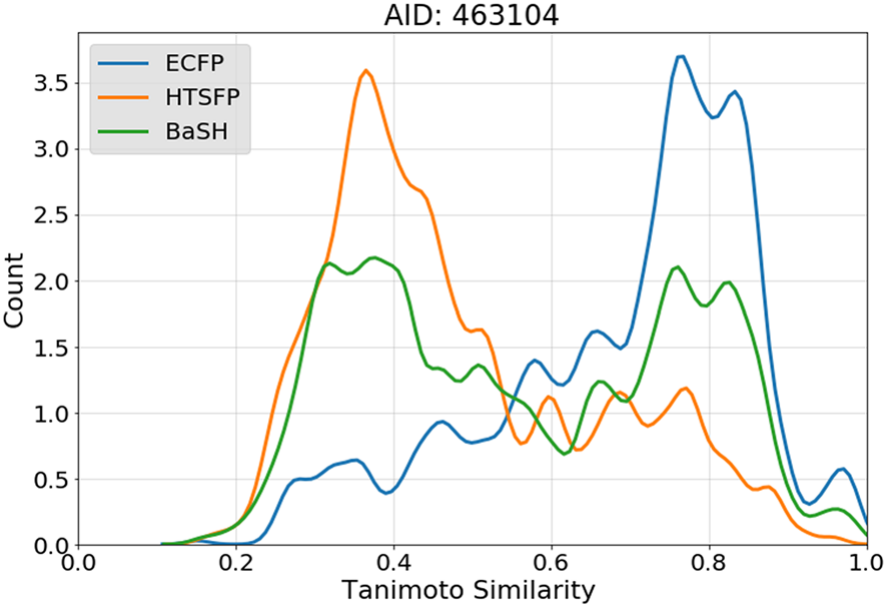
Compound diversity of top scoring 1000 compounds. The nearest neighbor Tanimoto similarity was calculated for each of the 1000 compounds and plotted as a fitted histogram. The nearest neighbor similarity was calculated for each of the 3 predictive models BaSH: green, HTSFP: orange, and ECFP4: blue

#### Nearest neighbor Tanimoto similarity

To further investigate the results shown in the Venn diagrams a nearest neighbor analysis was performed. The Venn diagrams revealed the presence of different scaffolds but did not reveal how different these scaffolds were to one another structurally. By plotting the nearest neighbor for each compound, the overall structural diversity of the compound set could be visualized. Figure 4 shows the plot of the nearest neighbor Tanimoto similarity of the top scoring 1000 compounds of assay 463104 using each of the three prediction models. The plot shows that the compounds predicted using the ECFP4 share a larger degree of structural similarity relative to the predictions made using the HTSFP. The majority of the compounds predicted using ECFP4 have a Tanimoto similarity between 0.7 and 0.9 whereas the majority of compounds predicted using the HTSFP have a Tanimoto similarity around 0.3–0.4. The compounds predicted using the BaSH fingerprint had similarity values in between those of ECFP4 and the HTSFP. This distribution provides evidence that the ECFP4 is not as well suited for scaffold hopping as the HTSFP or the BaSH. It must also be mentioned that the diversity of the ECFP predictions is highly dependent on the diversity of its training data, i.e. highly diverse training data can also lead to diverse predictions for ECFP but these predictions would theoretically never expand into new chemical space as well as bioactivity fingerprints. Although the compounds predicted with the BaSH fingerprint exhibit a lower degree of structural diversity than the HTSFP, the predictive accuracy of the BaSH is better and is therefore the favored model. All 24 test assays followed a similar trend as the seen in Fig. 4, plots for all test assays are shown in Additional file 1: Figure S4.

### Compound ranking comparison

**Fig. 5:**
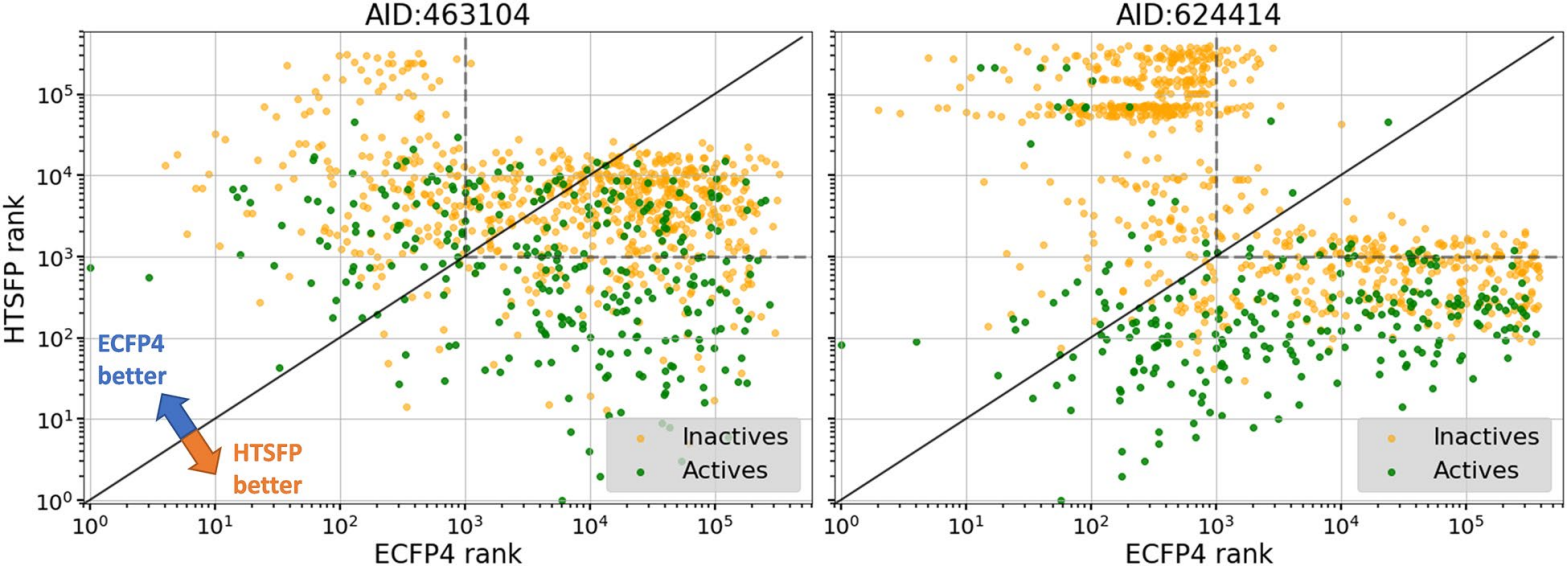
Comparison of compound rankings for the three prediction models. The top scoring 1000 compounds predicted using the BaSH are shown. The rankings of the same compounds in the HTSFP model (Y-axis) and the ECFP4 model (X-axis) are compared. The green and orange dots represent active and inactive compounds, respectively. The dashed line boarders the upper right quadrant, which refers to rankings outside the top 1000 rankings for the HTSFP and EFCP4. Results from two test assays are shown in the plots left: AID 463104 and right: AID 624414

The top ranking 1000 compounds predicted using BaSH fingerprint were selected. Compounds could be ranked based on their probability scores obtained from the random forest model. This probability score is based on consensus voting by the decision trees of the random forest model. The rankings of these compounds in the ECFP4 predictions were plotted against the rankings from the HTSFP predictions. These plots for assays 463104 and 624414 are shown in Fig. 5. The green dots represent active compounds and the orange dots represent inactive compounds. Compounds above the diagonal black line were ranked higher in the ECFP4 model and compounds below the line were ranked higher in the HTSFP model, i.e. a smaller number equals higher rank. The dashed lines represent the boundary for rankings not in the top 1000 for either the ECFP4 or HTSFP. It was expected that the top 1000 BaSH compounds would be within the top ranking 1000 compounds of either the ECFP4 or the HTSFP i.e. not in the upper right quadrant of the plot. This expectation would give a rise to an ‘L’ shaped clustering. This ‘L’ shaped clustering was only partially visible in the plot of assay 624414, but even here a small number of the compounds were located outside the expected rankings, i.e. in the upper right quadrant. The remaining 24 assays larger portions of the 1000 BaSH predicted compounds appeared in the upper right quadrant (see Additional file 1: Figure S6). For example, assay 463104 showed a large portion of compounds ranked outside the top 1000 for both the ECFP4 and the HTSFP. The fact that the BaSH fingerprint predicts many active compounds outside the top 1000 rankings of ECFP4 and HTSFP demonstrates a synergistic effect between structural and bioactivity descriptors. This synergistic effect allows for improved predictive performance and scaffold hopping capability. The scatter plots for all 24 test assays are show in the Additional file 1: Figure S6.

#### Feature importance

**Fig. 6:**
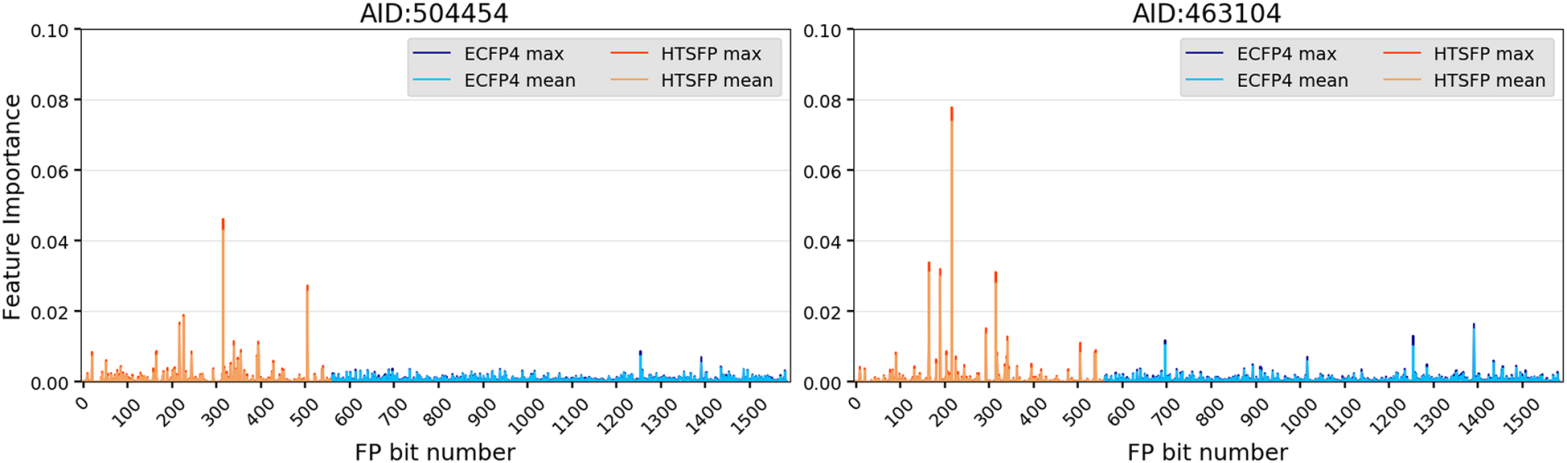
Feature importance of the combined fingerprint (BaSH) for two representative assays of the validation set. Features 0–560 correspond to the HTSFP portion (orange) and features 560–1584 correspond to the ECFP4 portion (blue) of the combined fingerprint. The light and dark shades of each feature refer to the mean and max values from the six-fold cross validation, respectively

The feature importance of each of the models for the BaSH fingerprint six-fold cross validation were analyzed using a feature importance function [23]. The feature importance for assay 463104 is plotted in Fig. 6. Features 0–560 refer to the HTSFP (orange) while features 560–1584 refer to the ECFP4 (blue). This plot shows the average and the maximum importance (light and dark shades respectively) calculated from the six-fold cross validation. The ECFP4 seldom shows any features that are significantly more important than others and in general displays an overall constant basal level of importance, i.e. almost every ECFP4 feature has some importance. In one case, assay 555, where the HTSFP had no significant contribution did some of the ECFP features show pronounced importance. For some of the assays certain features in the ECFP4 show higher importance but due to the way the ECFP4 is folded into a 1024 binary vector it is impossible to determine precisely which structural features each bit corresponds to. The HTSFP portion of the BaSH shows much greater variability in feature importance from assay to assay. Overall the basal level of feature importance in the HTSFP is lower than in the ECFP4, although a small number of the HTSFP features show highly pronounced importance values. This trend of pronounced HTSFP features could be seen across 19 of the 24 test assays (see plots in Additional file 1: Figure S7). The assays corresponding to these pronounced features were investigated in more detail and an overview of the top 5 most important HTSFP features for each test assay is shown in Additional file 1: Table S1. Discussed here are three representative test assays i.e. AID 798, AID 463104, and AID 504454. The assay biological targets corresponding to the top 5 most important HTSFP features were determined and are shown in Table 2.

**Table 2:** The PubChem assays corresponding to the five highest importance features as seen in Fig. 6

PubChem AID	Feature number	Importance value	Assay biological target
798	Test assay		Coagulation factor XI
800	532	0.044	Coagulation factor XIIa light chain
873	551	0.035	Human kallikrein 5 (hK5) serine protease
1046	17	0.020	Prothrombin
687	466	0.004	Coagulation factor XI
680	458	0.003	Coagulation factor XI

PubChem AID	Feature number	Importance value	Assay biological target
463104	Test assay		Activators of the adaptive arm of the unfolded protein response
485346	216	0.074	E3 ubiquitin-protein ligase Mdm2/MdmX
2732	165	0.031	Inhibitors of CHOP to regulate the unfolded protein response
449763	190	0.030	Activators of the apoptotic arm of the unfolded protein response
588405	315	0.028	Protein phosphatase 1 regulatory subunit 15A
540308	293	0.014	Melanocortin receptor 4 (MC4R)

PubChem AID	Feature number	Importance value	Assay biological target
504454	Test assay		Beta-2 adrenergic receptor
588405	315	0.043	Protein phosphatase 1 regulatory subunit 15A
743279	505	0.026	Inhibitors of inflammasome signaling: IL-1-beta
488899	226	0.018	MITF microphthalmia-associated transcription factor
485346	217	0.016	E3 ubiquitin-protein ligase Mdm2/MdmX
624352	394	0.010	Endothelial PAS domain-containing protein 1

Column one refers to the PubChem AID, column two refers to feature position with the combined fingerprint, column 3 indicates the importance value, and column 4 gives information on the assay target

Test assay 798, from the PubChem dataset, is a biochemical assay aimed at identifying compounds which inhibit coagulation factor XI. The random forest model used to make the retrospective predictions on this assay was analyzed and the feature importances were determined. According to the ROC AUC shown in Fig. 2 the HTSFP expressed better performance than the ECFP although the performance difference between the two was negligible when considering the MCC score. The five most important features all correlate to compounds which were active against targets involved in secondary hemostasis which all have a serine protease function. The 4th and 5th most important features referred to assays 687 and 680 which also targeted coagulation factor XI but had surprisingly low importance. Closer investigation of these two assays revealed the reason for their relatively low importance. One point was that the two assays only had tested compound sets with a compound overlap of 32511 and 59853 respectively, which is relatively small compared to the 798-test assay (218716 compounds). Another point was that the agreement between the assays was limited, only 23/94 and 21/120 actives were in agreement with the 798-test assay for assays 687 and 680 respectively. The fact that the random forest model is successfully identifying and correlating compounds which have similar activities against similar targets is an expected result. These results act as a form of validation for the methods and procedure used in this study.

Test assay 463104 is a cell-based, phenotypic assay targeted at identifying promoters of the unfolded protein response (UPR), specifically the adaptive arm. UPR is involved in protein degradation as well as apoptosis related processes. The top 5 most important features of the random forest model were determined for this assay and their corresponding assay biological targets are listed in Table 2. The first most important feature corresponds to an assay targeting E3 ubiquitin protein ligase. The E3 ubiquitin protein ligase is involved in Ubiquitination processes, which are directly involved with protein degradation, and are a vital element of the UPR. The 2nd and 3rd most important features both refer to assays also targeting different domains of the UPR. The 4th most important feature corresponds to an assay targeting ‘Protein phosphatase 1 regulatory subunit 15A’. This target is involved with regulation of protein synthesis and plays a role in the UPR, its relatively high importance suggests that this target is likely also present or closely related to the target(s) in the 463104-test assay. Again, the fact that the four most important features all correspond to compounds which were active in the UPR process, validates the method and observed results. The 5th most important feature corresponds to an assay targeted at melanocortin receptor 4 (MC4R). MC4R is a GPCR which has no known association to the UPR. This result suggests that the predictive model has the ability to draw correlations from unrelated features of the HTSFP, thereby supporting a wider applicability domain which has also been observed in previous studies for HTSFPs [5–7]. Considering this lack of correlation between the two targets, it must also be mentioned that the relative importance of this feature is much lower (0.013) as can be inferred from Fig. 6.

Assay 504454 is a cell-based, phenotypic assay aimed at identifying inhibitors of the beta-2 adrenergic receptor (b2AR) which is a member of the GPCR family. The biological target of test assay 504454 did not have any known relation to the biological targets of the top 5 features. The PubChem assays and their associated biological targets corresponding to these five features are listed in Table 2. These five assays target a variety of different functional proteins, none of which are members of the GPCR family. The targets types include regulatory subunits, inflammasomes, protein ligases, and two transcription factors. This result shows activity predictions for a given assay are not dependent on the HTSFP containing assays with related or similar biological targets. In other words, valid activity predictions can be made for compounds which are being tested on previously unexplored targets, as has shown in previous studies [5–7].

## Conclusion

From analysis of the various metrics used to assess the prediction quality of the BaSH fingerprint it can be concluded that the BaSH yields a viable improvement in prediction performance relative to the individual ECFP4 and HTSFP. The MCC, F1 score, enrichment factor, ROC-AUC and Cohen’s kappa score all show evidence of the combined fingerprint’s enhanced performance. The results indicate that this combined fingerprint is a useful tool for scaffold hopping, detecting not only a more diverse set of active compounds with different scaffolds but also identifying novel scaffolds that were not identified with either the ECFP4 or the HTSFP. The improved scaffold hopping ability of the BaSH fingerprint was further supported by the nearest neighbor analysis. A comparison of the compound rankings provided evidence of the synergistic effects between the structural and bioactivity-based fingerprints. Feature importance analysis quantified the relative contributions of ECFP4 and HTSFP to the BaSH predictions, revealing that a small subset of the HTSFP features contribute most to the overall performance. This subset of features often corresponded to assays with targets biologically related to the test assays, however, this was not necessary for the HTSFP’s increased contribution. Naturally, the BaSH fingerprint has some limitations due to its HTSFP portion. The most notable limitation being the availability of historical HTS data, meaning that only previously tested compounds can be used. Furthermore, the presence of assays in the HTS portion which have related targets to the test assay has a strong positive influence on model performance. Therefore the inverse also applies, models built for previously untested targets or targets with no close relations, may exhibit reduced performance, although meaningful predictions are still possible. An example for this is provided by assay 504454 for the beta-2 adrenergic receptor. Although the top 5 most important features correlated with unrelated targets, predictive performance was reasonable, with ROC-AUC of 0.78 for HTSFP compared to 0.70 for the ECFP4. Another point is that assays with only small numbers of ‘active’ samples which have limited diversity don’t contain sufficient information for training of a reliable model, which has a negative effect on the model performance. A positive characteristic of the BaSH fingerprint is that although the HTSFP portion was very sparse, and contains a lot of noise through the labelling of missing data points as ‘inactive’, the noise did not negatively effect on the overall performance. Due to the relatively small size of the HTSFP to the ECFP4 (561 to 1024 respectively), the noise presented by the missing data is possibly drowned out by dominating presence of the ECFP. This provides a point of additional optimization, by applying weighting factors or altering the length of the ECFP further performance increases could potentially be achieved. Another positive characteristic of the BaSH is that compounds with sparse or no HTSFP fingerprints need not be filtered out, as they are fortified by the presence of the ECFP and therefore valid activity predictions can still be made. An alternative approach would be to use continuous data such as IC50 values and apply an adjustable threshold to optimize the HTSFP to possibly get further model improvements. Additional improvements could possibly be achieved by implementing frequent hitter or interference compound filters. Overall the BaSH fingerprint appears to be a promising tool for activity prediction and provides evidence that combining different types of descriptors is a valid method for boosting model performance.

## Methods and data

### Dataset

For this research 24 HTS assays obtained from PubChem were investigated retrospectively, they contained diverse ratios of active to inactive compounds as well as varying target types and a range of assay sizes ranging from approximately 20,000–400,000 compounds per assay. An overview of the 24 test assays is shown in Table 1.

### Descriptors for models

#### Generation of HTS fingerprints

A set of 582 HTS assays were downloaded from the PubChem database. Assays containing fewer than 20,000 compounds were discarded, leaving a total of 561 assays. This cut off was made to reduce the size and sparsity of the HTS fingerprint. The ‘Active’ or ‘Inactive’ activity flags set by PubChem were used to build the fingerprint. If any compounds were tested multiple times with mixed activity outcomes, the most common activity flag was used. In the case where there were equal numbers of active and inactive flags, the active flag was used. All compound’s activity flags were collated into a matrix of ‘compound ID’ versus ‘Assay ID’, with dimensions 715,328 (compounds) × 561 (assays). The fingerprint was subsequently binarized by converting all ‘active’ labels to ‘1’ and ‘inactive’ labels to ‘0’. All missing data was also set to ‘0’, the reasoning for this was that the HTS data is very unbalanced and a compound with unknown activity has a much higher probability of being inactive and is therefore given the label of an inactive bit. Each test assay was removed from the HTSFP prior to its analysis.

#### Structural descriptors

For the same list of 715,328 compounds as in the HTSFP, ECFP4 fingerprints were created. The PubChem HTS data contained only the CID for the compounds and to make the ECFP fingerprints the smiles for each compound was required. Using the list of CIDs, the Smiles for each compound were downloaded from the PubChem database. The Morgan circular fingerprint (an analogue of ECFP) implemented in RDKit was used [24]. After removal of compounds with invalid or unreadable smiles for RDKit, a compound set of 715327 was obtained. The bit length was set to 1024 bits and the fragment radius was set to 2 (diameter 4). Tests were run comparing 1024-bit ECFP4 with 1024-bit ECFP6 for one of the test assays. Only minor differences could be seen in predictive performance but the ECFP6 appeared to be slightly weaker, therefore the ECFP4 was chosen for the full analysis. The performance of the 1024 bit ECFP4 was compared with a 2048 bit ECFP4, the results showed no noticeable improvement when using the longer ECFP4, therefore the shorter version was chosen.

#### Generation of the BaSH fingerprint

The bioactivity-structure hybrid (BaSH) fingerprint was created by concatenating the ECFP4 to the HTSFP, giving a new fingerprint of length 1585 (561 + 1024). These fingerprints were created using the same compound set (715327) as output from the ECFP4. Prior to analysis of each test assay, it was first removed from the BaSH fingerprint.

### Modelling methods

Due the nature of the random forest learning method, where specific features within a fingerprint are identified and not the entire fingerprint, it was theorized that RF would be the best suited technique to deal with the large portion of majorly sparse HTSFPs in the dataset. A test run was performed comparing random forest with support vector machine models of the Scikit-learn package [23]. The two models were tested on one of the ten test assays, the random forest showed better performance according to the ROC AUC values and also ran significantly faster.

The random forest classifier machine learning package from Scikit-learn was used for building models of three different descriptor types, i.e. ECFP4, HTSFP, and BaSH. Here the ECFP4 and HTSFP were used for comparative and benchmarking purposes in all performance evaluations. The hyperparameters were optimized on a set of 10 randomly chosen assays, see Table 3 for assay information. For testing each assay was removed from the training data of the HTSFP and BaSH. An independent hyperparameter grid search was carried out for each of the three descriptor types. The most commonly occurring hyperparameter setting across the 10 test assays was chosen. The optimized hyperparameters were as follows:

**Table 3:** Overview of test assays used in hyperparameter search

AID	Compounds tested	Actives	% Actives	Target information	Assay type
834	84880	123	0.14%	Potentiators of clotrimazole	Cell-based
1236	218607	799	0.37%	Calpain II inhibitors	Biochemical
1510	217964	569	0.26%	Sphingosine-1-phosphate receptor 4 (S1P4)	Cell-based
1899	302667	998	0.33%	Hepatitis C Virus (HCV) core protein	Biochemical
2732	218659	8240	3.77%	DNA damage-inducible transcript 3—CHOP—regulates UPR	Cell-based
463165	305614	1365	0.45%	Regulator of G-protein signaling 4 isoform 2 (RGS4)	Cell-based
588621	359231	887	0.25%	Tyrosine-protein phosphatase non-receptor type 5	Biochemical
602229	362013	1281	0.35%	Photoreceptor-specific nuclear receptor (NR2E3)	Cell-based
720543	369939	2005	0.54%	Alpha/beta hydrolase domain containing protein 4 (ABHD4)	Biochemical
1117267	91911	1155	1.26%	Activators of Transthyretin (TTR) transcription	Cell-based

Columns represent PubChem AID, number of compounds tested in assay, number of actives, percentage of actives, target information, and assay type

*HTSFP: n_jobs *= −*1, n_estimators *= *150, class_weight *= *‘balanced’, max_features *= *‘sqrt’, criterion *= *‘entropy’, max_depth *= *40, min_samples_split *= *2, min_samples_leaf *= *5, random_state *= *56*

*ECFP4: n_jobs *= −*1, n_estimators *= *200, class_weight *= *‘balanced’, max_features *= *‘sqrt’, criterion *= *‘gini’, max_depth *= *30, min_samples_split *= *2, min_samples_leaf *= *8, random_state *= *56)*

*BaSH: n_jobs *= −*1, n_estimators *= *150, class_weight *= *‘balanced’, max_features *= *‘sqrt’, criterion *= *‘gini’, max_depth *= *None, min_samples_split *= *2, min_samples_leaf *= *8, random_state *= *56)*

The number of trees (n_estimators) was set to 150/200 as above this threshold model performance did not show noticeable improvement. A ‘balanced’ class weighting was used due to the imbalanced nature of the data, the ‘balanced’ setting of this hyperparameter was vital for adequate performance of the models. For the purpose of reproducibility a random_state = 56 was used. For model validation a sixfold cross-validation was performed, averages and standard deviations were calculated across the six folds for each of the test assays. A stratified sampling method was used to generate each fold, this meant that each fold had the same ratio of ‘active’ to ‘inactive’ samples. The metrics for each test assay were calculated using the mean values and standard deviations calculated across the six folds.

For the scaffold hopping analysis the true positives in the top ranked 1% of predictions were extracted for each cross-validation fold and their compound IDs (CIDs) were mapped to smiles. Using RDKit each compound was converted to a topological Bemis-Murcko scaffold (generic scaffold) i.e. all side chains were removed, all heteroatoms converted to carbons, and all bond orders set to 1 (all C = sp3). The number of unique topological scaffolds were then counted and averaged across the six folds. The unique scaffolds predicted from each of the 3 tested fingerprints were compared using Venn diagrams made from the matplotlib-venn add-on. Venn diagrams were made for each cross-validation fold and the average for each region in the diagram was taken to make the final diagram.

To compare the compound diversity for the predictions made using each of 3 fingerprint types (HTSFP, ECFP4, BaSH) a nearest neighbor comparison was performed. The nearest neighbor is calculated by performing a Tanimoto similarity comparison of the ECFP4 s for each compound in the prediction set. A Tanimoto similarity score of 1.0 is obtained for two compounds whose fingerprints are identical, whereas a score of 0.0 means that the fingerprints have no overlap. The similarity scores for all compounds in the top 1000 predictions were calculated and their distribution plotted (Fig. 4).

### Calculation of metrics

Receiver operator characteristic curves were constructed using the false positive rate (FPR) and true positive rate (TPR) while changing the classification threshold according to the prediction probability scores, this was performed using the SKlearn metrics library. The two equations in (1) show how the FPR and TPR are calculated.

1 $${\text{FPR}} = \frac{\text{FP}}{{{\text{FP}} + {\text{TN}}}}\quad {\text{TPR}} = \frac{\text{TP}}{{{\text{TP}} + {\text{FN}}}}$$

The precision and recall were calculated using the formulas shown in (2). The F1 score is the harmonic mean of the precision and recall and the calculation formula is also shown in (2).

2 $${\text{Precision}} = \frac{\text{TP}}{{{\text{TP }} + {\text{FP}}}}\quad {\text{Recall}} = \frac{\text{TP}}{{{\text{TP }} + {\text{FN}}}}\quad {\text{F}}1 {\text{score}} = \frac{{2{\text{TP}}}}{{2{\text{TP}} + {\text{FP}} + {\text{FN}}}}$$

The Matthews correlation coefficient (MCC) is a performance metric optimized for imbalanced datasets. The equation to calculate the MCC is shown in (3). The MCC covers a range from − 1 to 1, where a value of 1 indicates a perfect prediction, − 1 a perfect inverse prediction and 0 indicating prediction no better than random.

3 $${\text{MCC}} = \frac{{{\text{TP}} \cdot {\text{TN}} - {\text{FP}} \cdot {\text{FN}}}}{{\sqrt {\left( {{\text{TP}} + {\text{FP}}} \right)\left( {{\text{TP}} + {\text{FN}}} \right)\left( {{\text{TN}} + {\text{FP}}} \right)\left( {{\text{TN}} + {\text{FN}}} \right)} }}$$

The equation in (4) shows how the Cohen’s Kappa score is calculated, where $p_{o}$ is the relative observed agreement of a class (accuracy) and $p_{e}$ is the hypothetical probability of chance agreement. A kappa score of 0 reflects a performance no better than random chance, the more positive the score the better.

4 $$\kappa = \frac{{p_{o} - p_{e} }}{{1 - p_{e} }}$$

The Enrichment factor provides a measure of how much the model performance improves compared to random screening. The resulting score refers to a factor of improvement, where a score of 1.0 is equivalent to random. The formula to calculate the enrichment factor for the top scoring 1% of compounds is shown in (5). The Hitrate^1%^ refers to the rate of true positives in the top scoring 1%, and the Hitrate^100%^ refers to the hit rate for the overall screen.

5 $${\text{EF}}_{{1{\text{\% }}}} = \frac{{{\text{Hitrate}}^{{1{\text{\% }}}} }}{{{\text{Hitrate}}^{{100{\text{\% }}}} }}$$

Software used: Python 3.6.5, SKLearn 0.19.1, SciPy 1.1.0, RDKit 2018.03.1.0.

## Additional file

**Additional file 1.** Additional figures and table.

## Data Availability

The list of PubChem assays used is provided as a.txt file in Additional material, or the raw PubChem HTS files can accessed at https://figshare.com/articles/pubchemAssaysRAW_zip/7800554. Additional figures are also provided in an additional docX file. All source code is available from GitHub repository: https://github.com/oml90/Combining-Structural-and-Bioactivity-descriptors.

## Abbreviations

HTS: high throughput screening
BaSH: bioactivity-structure hybrid
ECFP: extended connectivity fingerprint
HTSFP: high throughput screening fingerprint
GPCR: G-protein coupled receptor
MCC: Mathews correlation coefficient
EF: enrichment factor
MoA: mechanism of action

## References

[1] Wu Y, Wang G (2018). Machine learning based toxicity prediction: from chemical structural description to transcriptome analysis. Int J Mol Sci.

[2] Simm J, Klambauer G, Arany A (2018). Repurposing high-throughput image assays enables biological activity prediction for drug discovery. Cell Chem Biol.

[3] Kauvar LM, Higgins DL, Villar HO (1995). Predicting ligand binding to proteins by affinity fingerprinting. Chem Biol..

[4] Fliri AF, Loging WT, Thadeio PF, Volkmann RA (2005). Biological spectra analysis: Linking biological activity profiles to molecular structure. Proc Natl Acad Sci..

[5] Petrone PM, Simms B, Nigsch F (2012). Rethinking molecular similarity: comparing compounds on the basis of biological activity. ACS Chem Biol.

[6] Riniker S, Wang Y, Jenkins JL, Landrum GA (2014). Using information from historical high-throughput screens to predict active compounds. J Chem Inf Model.

[7] Wassermann AM, Lounkine E, Urban L (2014). A screening pattern recognition method finds new and divergent targets for drugs and natural products. ACS Chem Biol.

[8] Muthas D, Boyer S (2013). Exploiting pharmacological similarity to identify safety concerns—listen to what the data tells you. Mol Inform..

[9] Wassermann AM, Lounkine E, Glick M (2013). Bioturbo similarity searching: combining chemical and biological similarity to discover structurally diverse bioactive molecules. J Chem Inf Model.

[10] Paricharak S, Bender A, Nigsch F, Nigsch F (2016). Analysis of iterative screening with stepwise compound selection based on novartis in-house HTS data. ACS Chem Biol.

[11] Campillos M, Kuhn M, Gavin AC (2008). Drug target identification using side-effect similarity. Science..

[12] Paull KD, Shoemaker RH, Hodes L (1989). Display and analysis of patterns of differential activity of drugs against human tumor cell lines: Development of mean graph and COMPARE algorithm. J Natl Cancer Inst..

[13] Weinstein JN, Kohn KW, Grever MR (1992). Neural computing in cancer drug development: predicting mechanism of action. Science..

[14] Wassermann AM, Lounkine E, Davies JW (2015). The opportunities of mining historical and collective data in drug discovery. Drug Discov Today..

[15] Mayr LM, Bojanic D (2009). Novel trends in high-throughput screening. Curr Opin Pharmacol.

[16] Battersby BJ, Trau M (2002). Novel miniaturized systems in high-throughput screening. Trends Biotechnol.

[17] Karnachi PS, Brown FK (2004). Practical approaches to efficient screening: information-rich screening protocol. J Biomol Screen.

[18] Glem RC, Bender A, Arnby CH (2006). Circular fingerprints: flexible molecular descriptors with applications from physical chemistry to ADME. IDrugs.

[19] Avram S, Bora A, Halip L, Curpan R (2018). Modeling kinase inhibition using highly confident data sets. J Chem Inf Model.

[20] Sturm N, Sun J, Vandriessche Y (2018). Application of bioactivity profile based fingerprints for building machine learning models. J Chem Inf Model..

[21] Helal KY, Maciejewski M, Gregori-Puigjane E (2016). Public domain HTS fingerprints: design and evaluation of compound bioactivity profiles from pubchem’s bioassay repository. J Chem Inf Model.

[22] Cortes Cabrera A, Petrone PM (2018). Optimal HTS fingerprint definitions by using a desirability function and a genetic algorithm. J Chem Inf Model.

[23] Pedregosa F (2011). Scikit-learn: machine learning in python %J. J. Mach. Learn. Res..

[24] Landrum GA (2018) RDKit: open source cheminformatics. http://www.rdkit.org

