# Supplementary material for: Combining structural and bioactivity-based fingerprints improves prediction performance and scaffold hopping capability
Source: J Cheminform. 2019 Aug 8;11:54. doi: 10.1186/s13321-019-0376-1 (PMC6686534; doi:10.1186/s13321-019-0376-1)
Supplement: Supplementary file 1 — Additional file 1. Additional figures and table. [file 13321_2019_376_MOESM1_ESM.docx]

# Additional Information

Databases

PubChem HTS assays. List of assays used for HTSFP generation attached: Pubchem_assay_list.txt


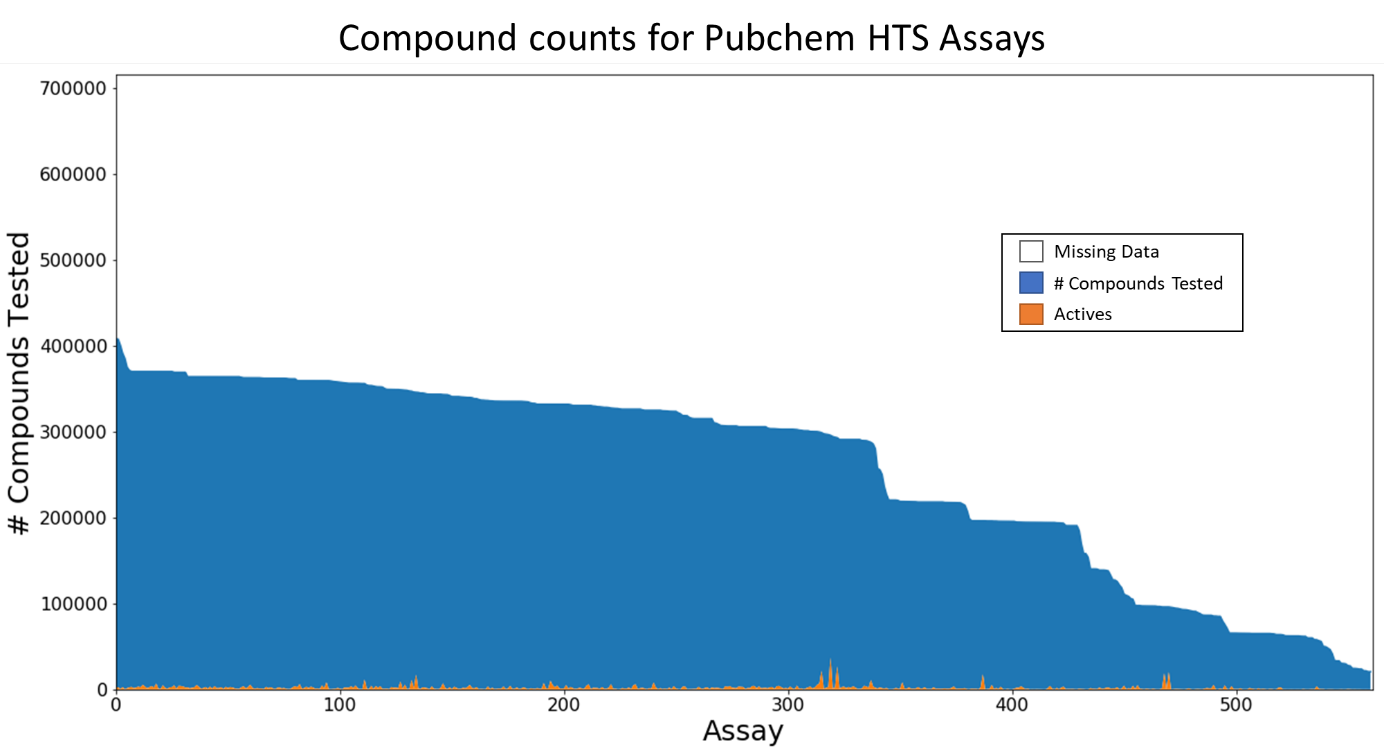
Figure S1. Overview of PubChem HTS data. 582 unique assays were used, and 715,326 unique compounds were identified. White space refers to the number of compounds not tested for a given assay (missing data), blue space refers to the count of compounds marked as ‘not active’, and orange space refers to the count of compounds marked as ‘active’.


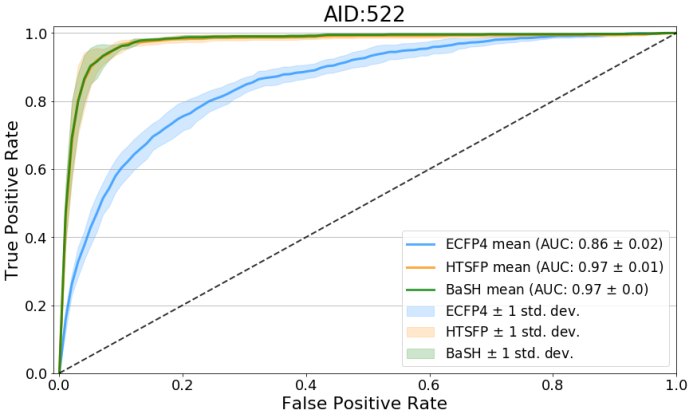

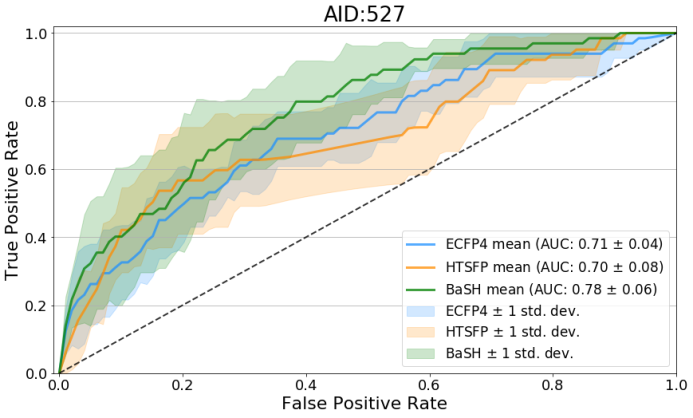

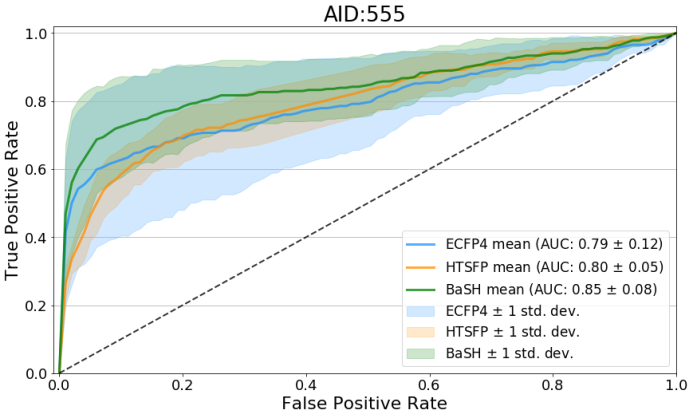

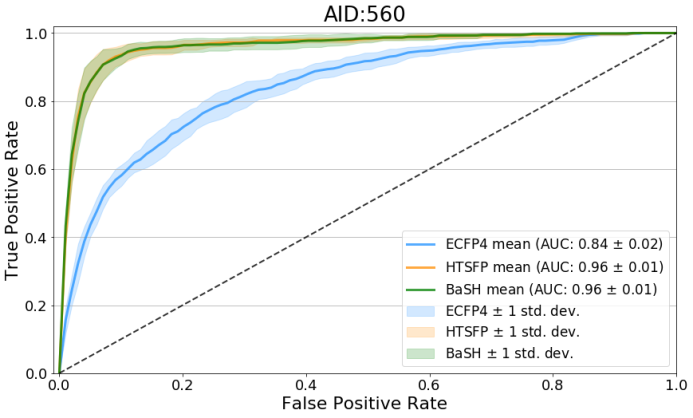

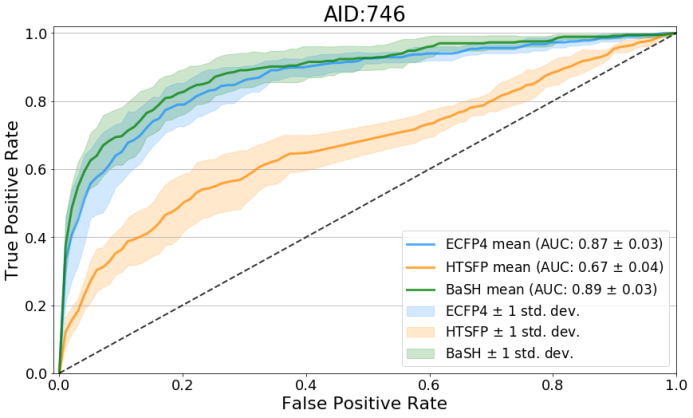

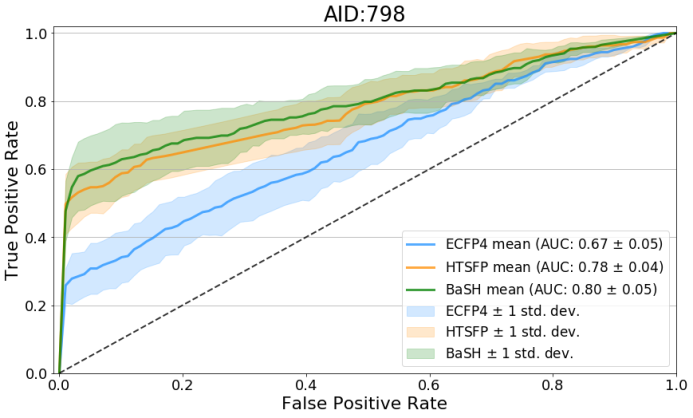

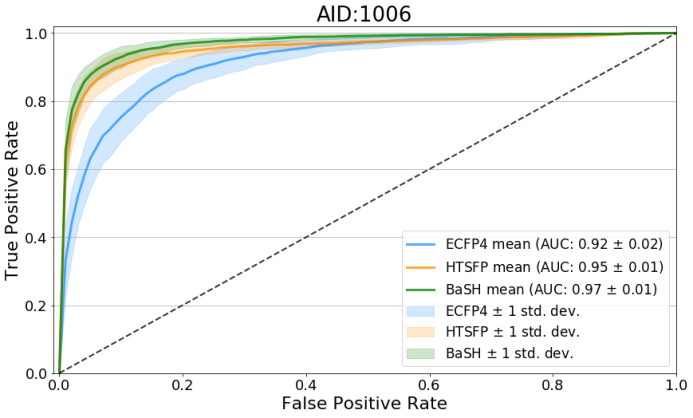

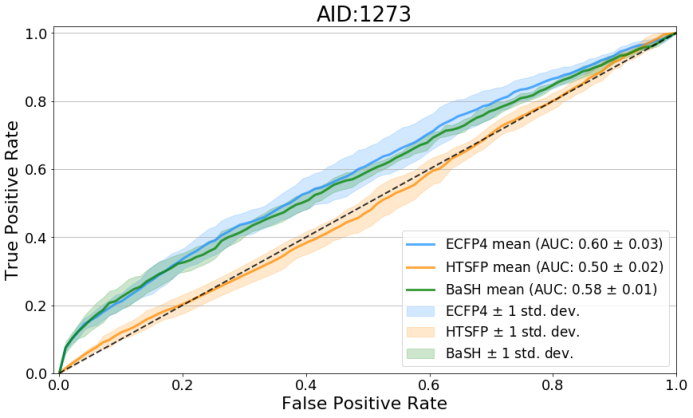

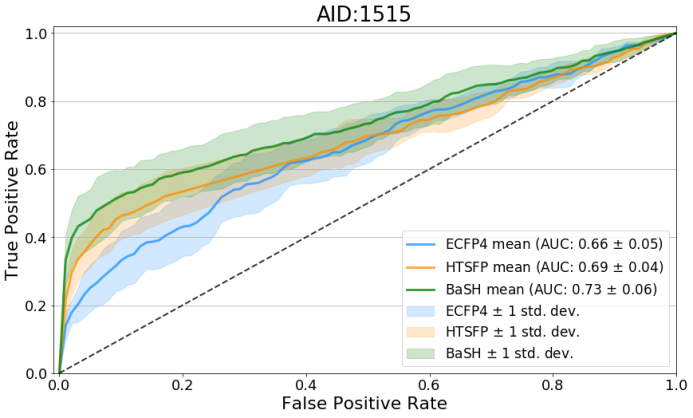

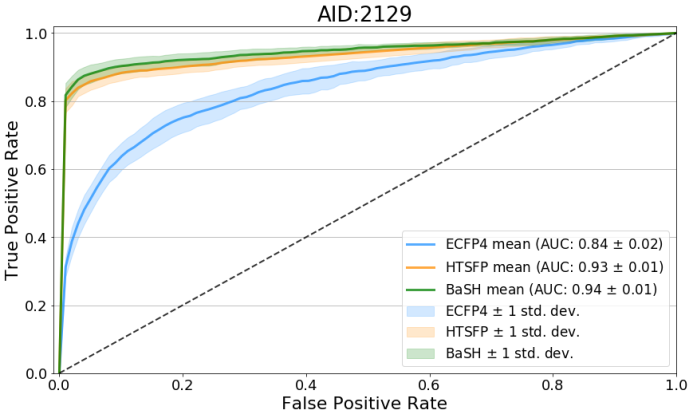

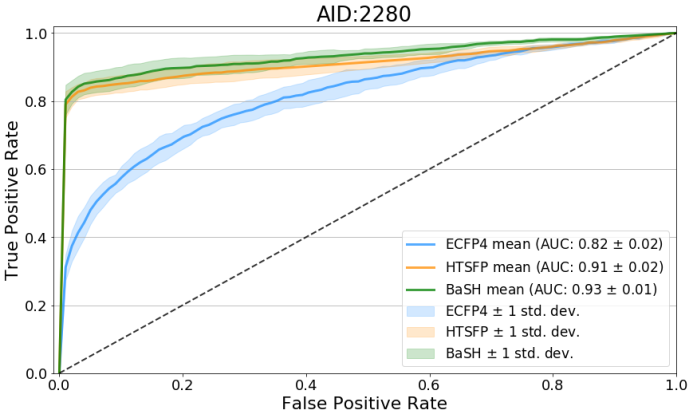

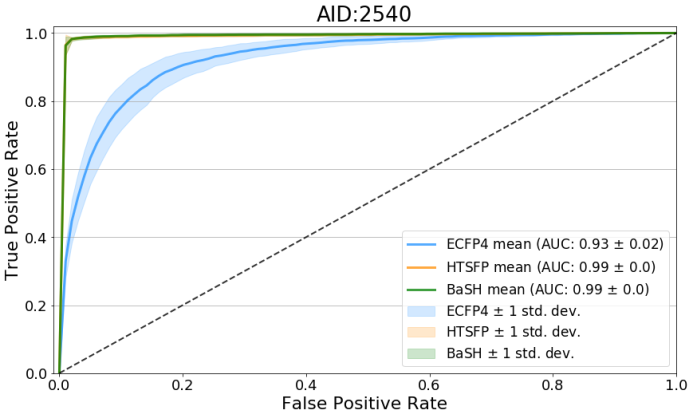

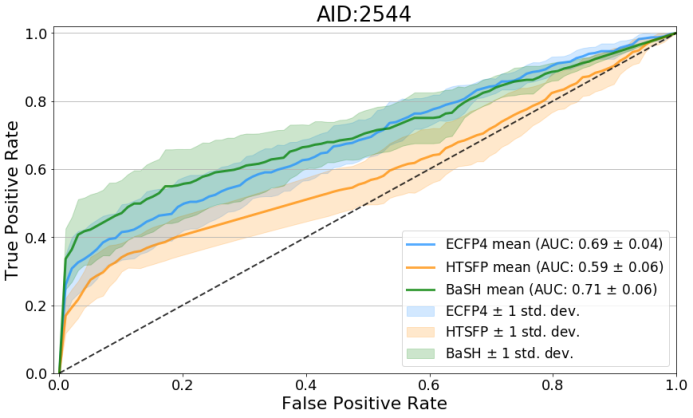

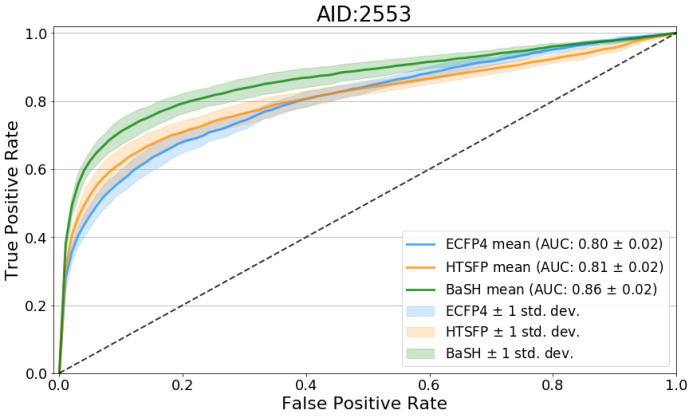

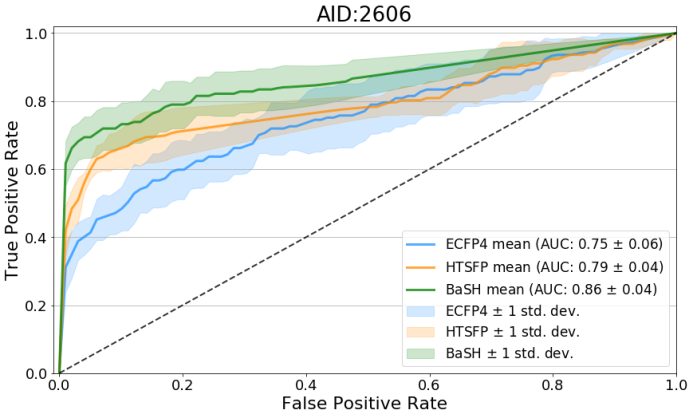

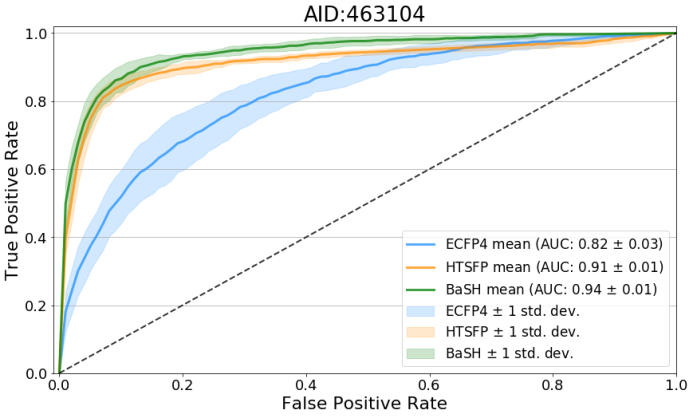

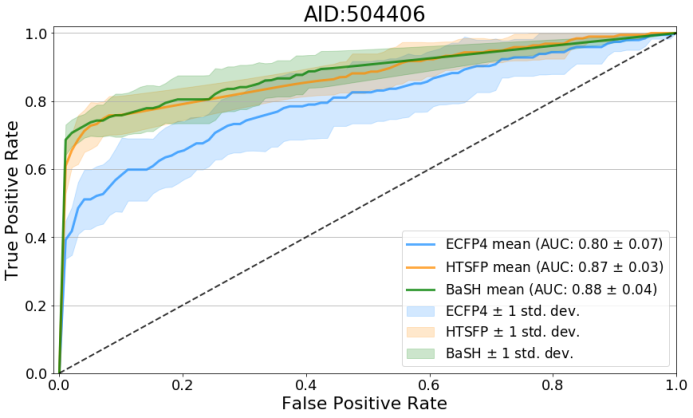

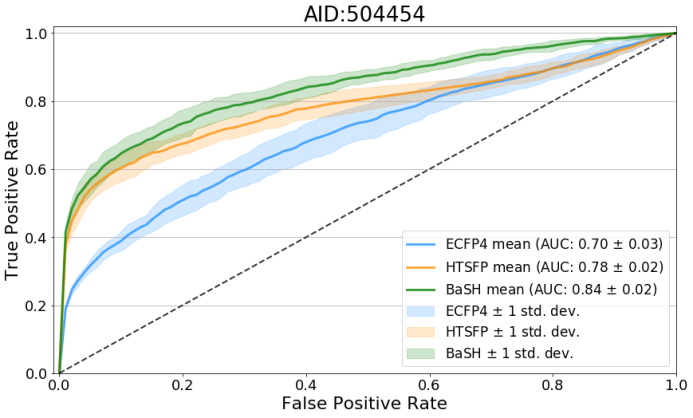

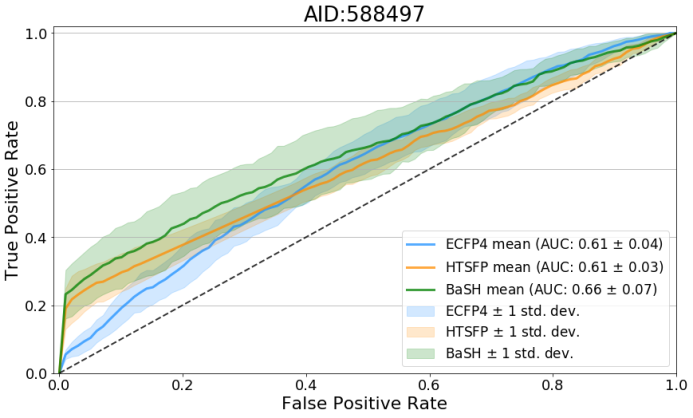

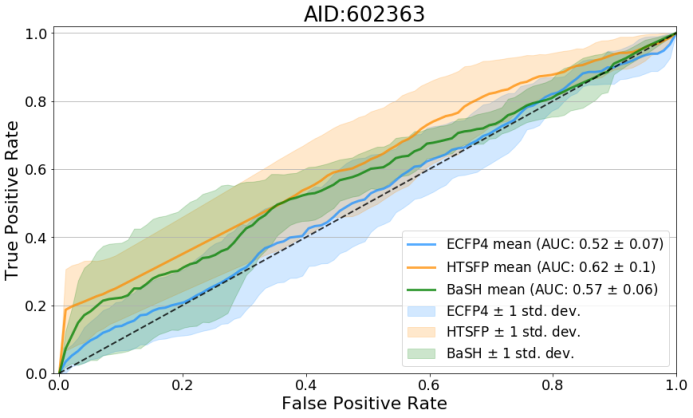

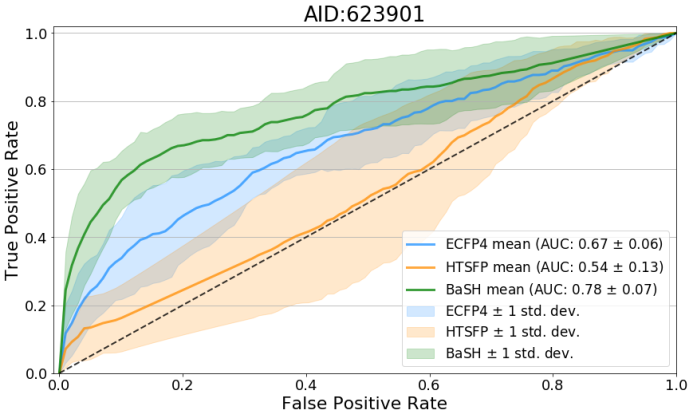

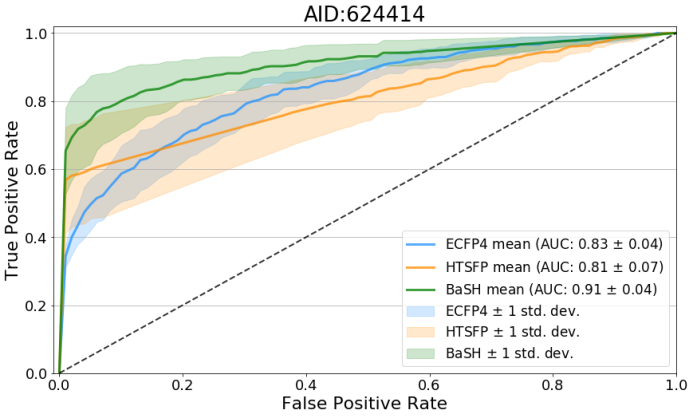

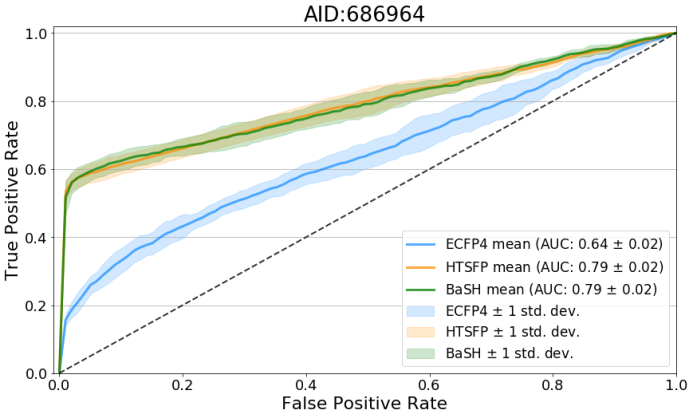

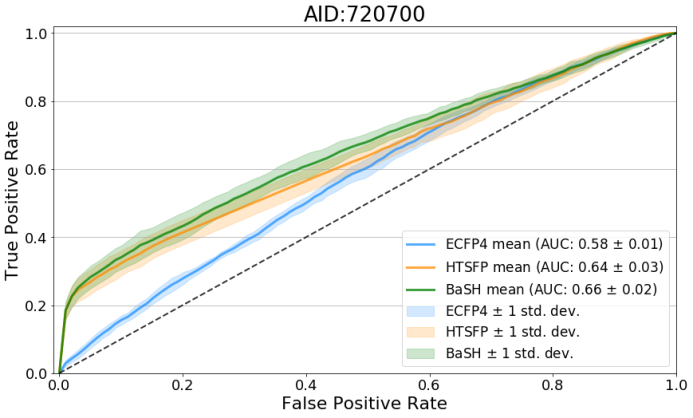


Figure S2. ROC curves of the 25 assays in the validation set, shown are the results of the random forest predictions for the three different descriptor types, ECFP, HTSFP, and BaSH (blue, orange, and green respectively). The solid line and the shaded area represents the


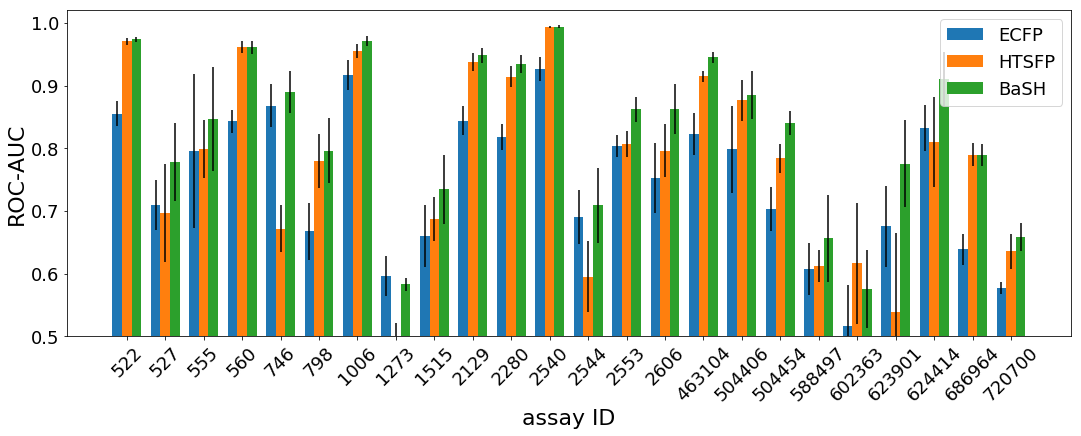

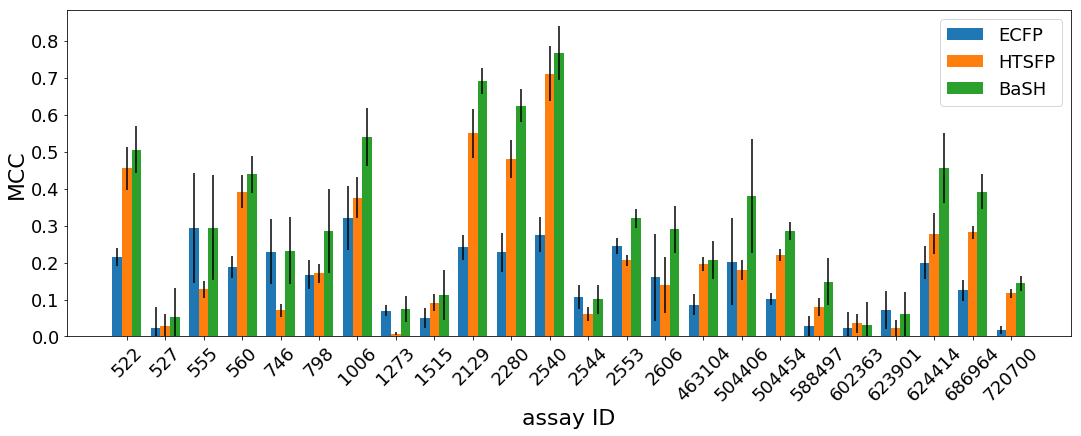

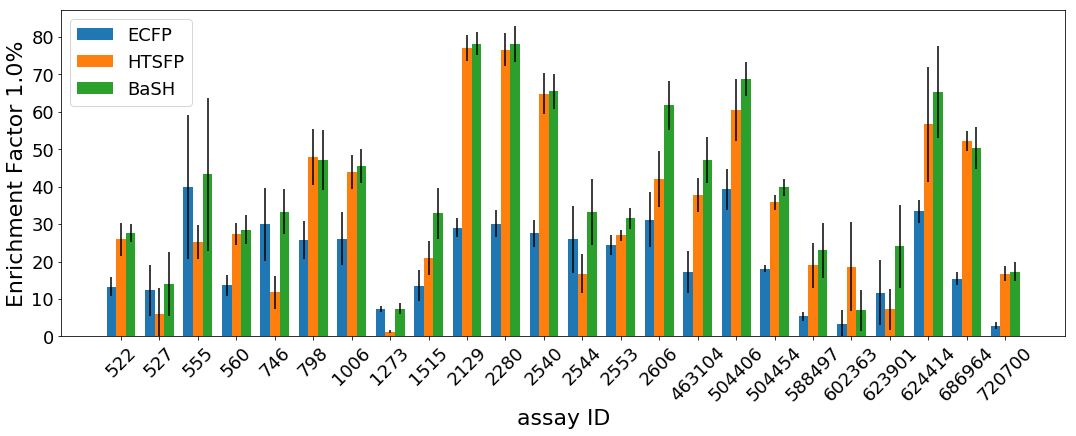

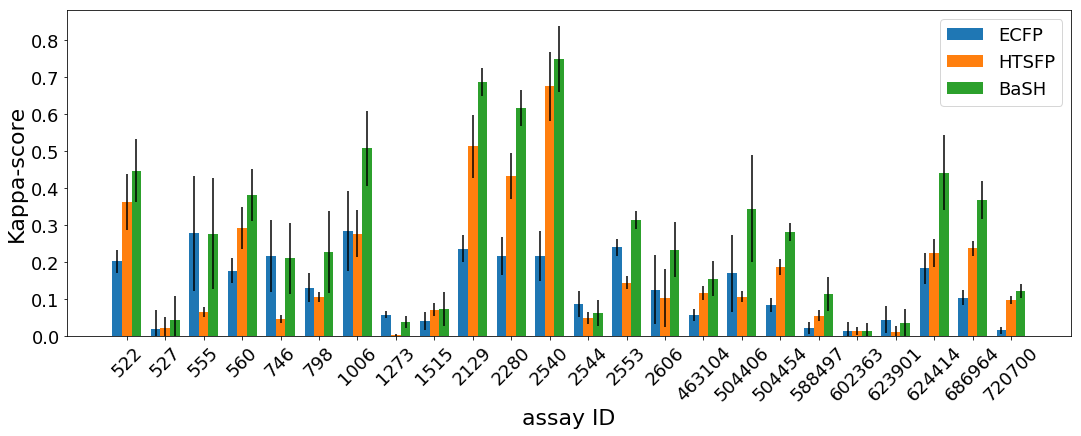

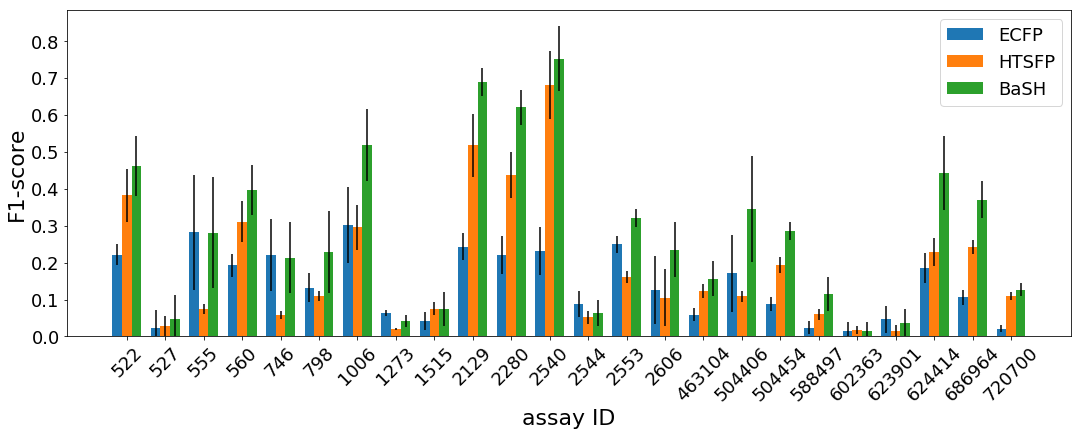

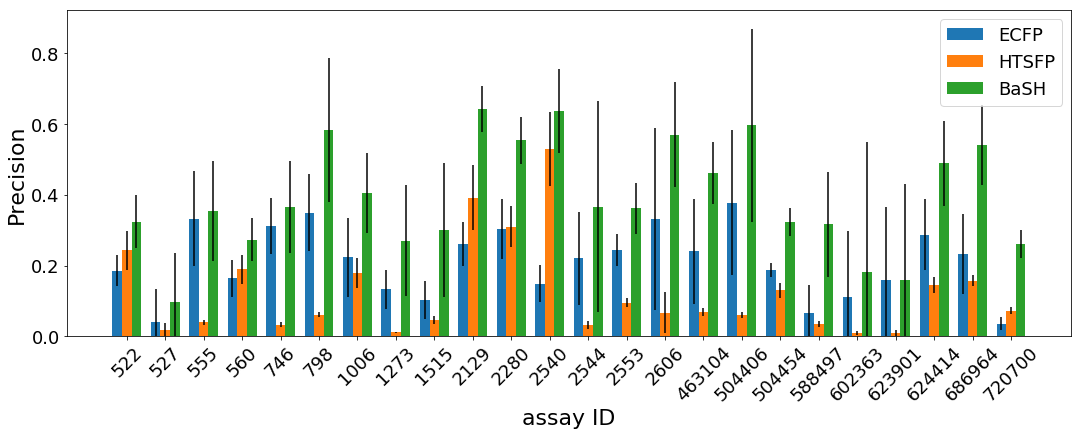

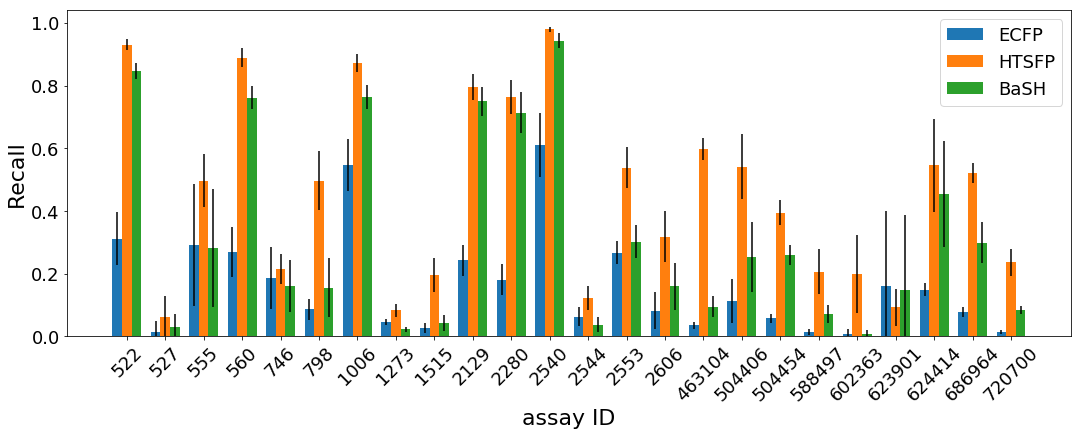
Figure S3. Bar plots of performance metrics for each of the 25 validation set assays. ROC AUC, Matthews’s correlation coefficient, Kappa score, F1-score, Precision, Recall, and enrichment factors.


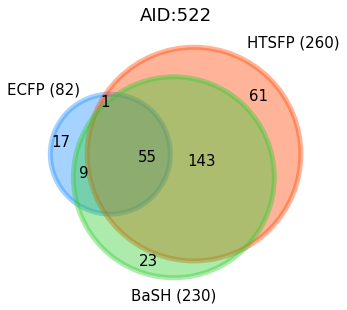

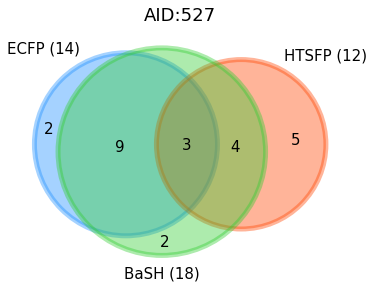

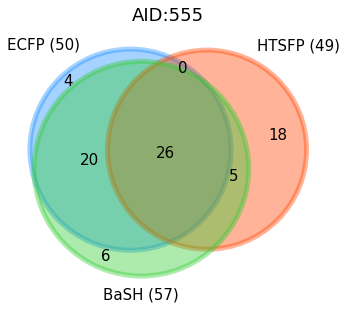

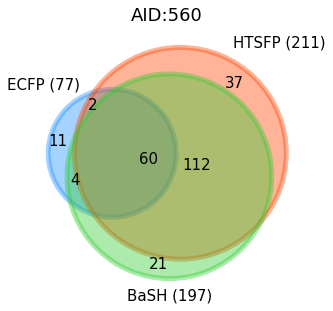

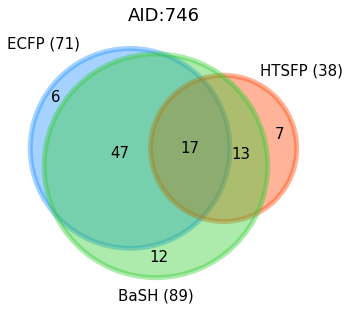

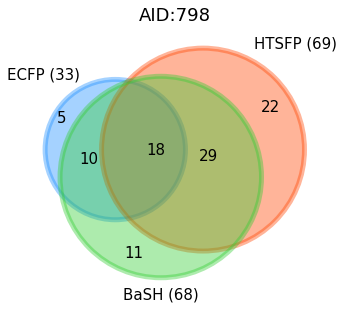

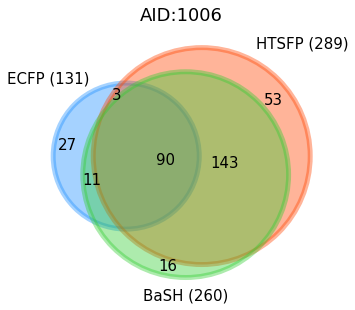

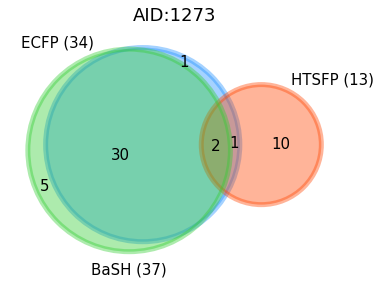

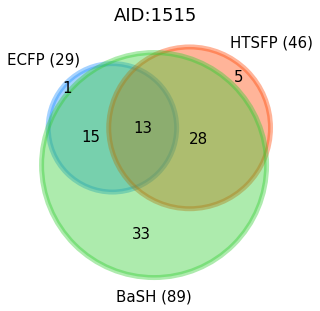

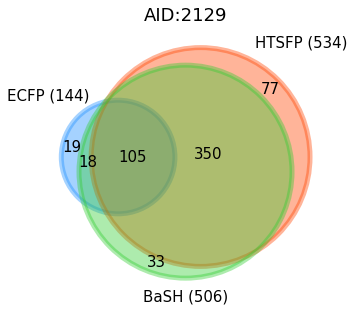

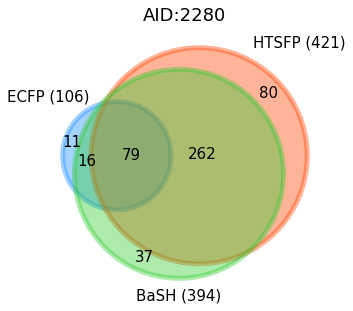

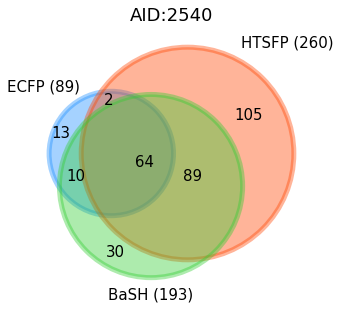

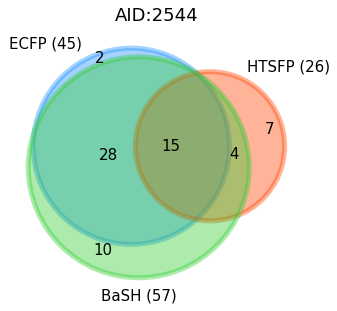

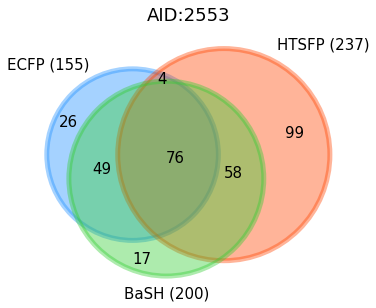

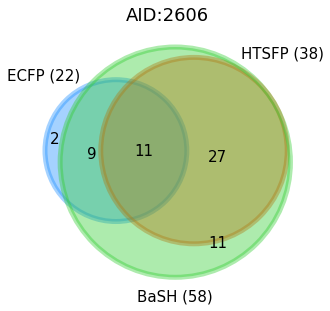

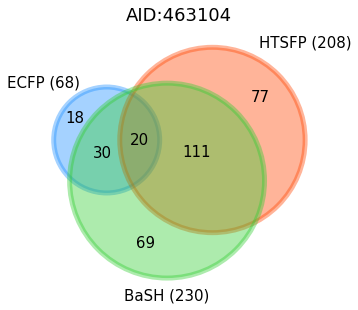

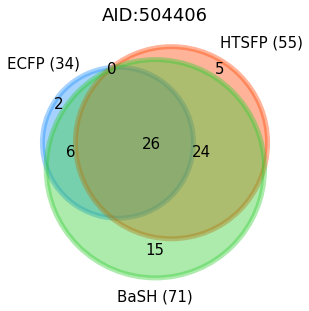

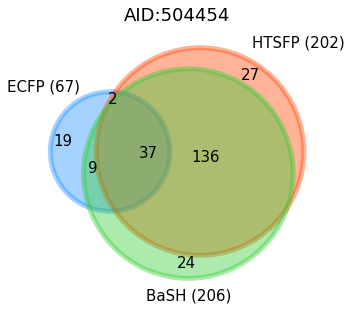

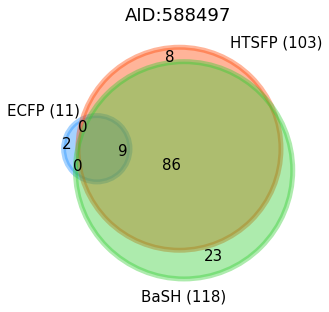

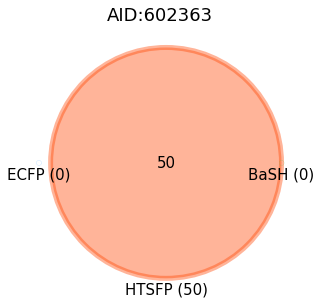

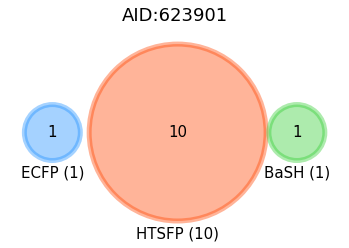

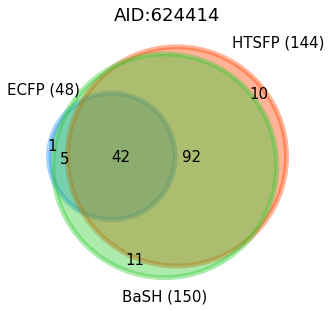

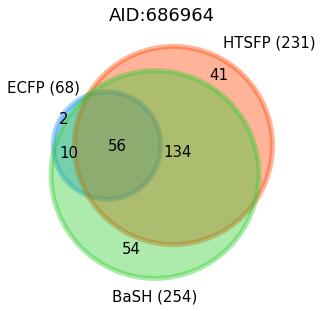

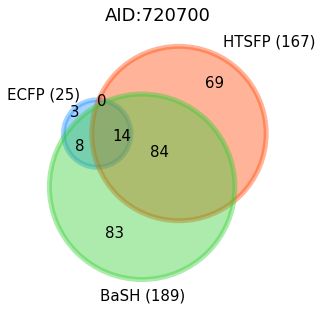
 Figure S4. Venn diagrams showing the number of unique topological scaffolds in the top scoring 1% of predictions. Each circle represents one of the three predictive models: BaSH, HTSFP, and ECFP4 (green, orange, blue respectively).


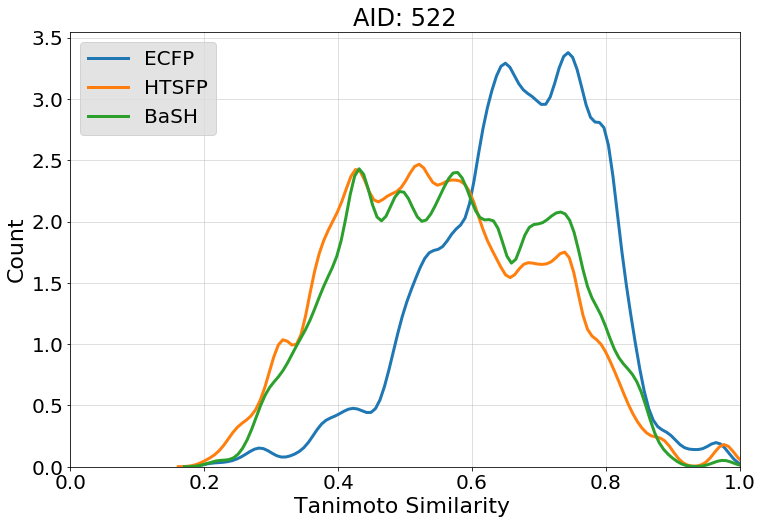

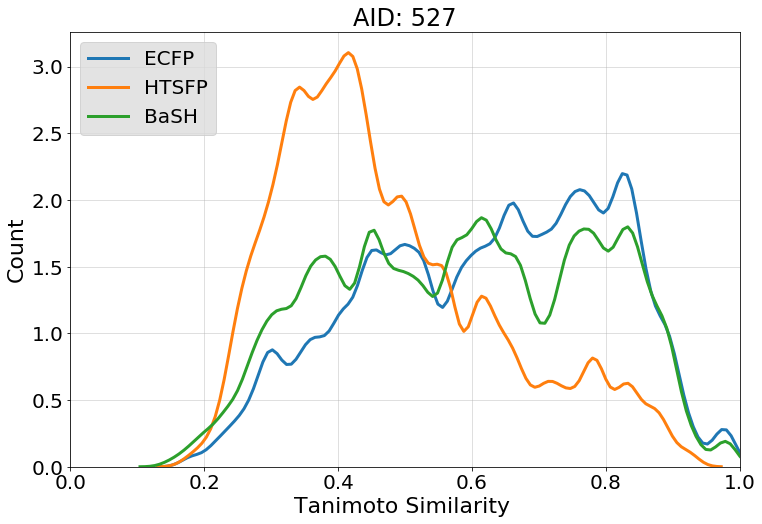

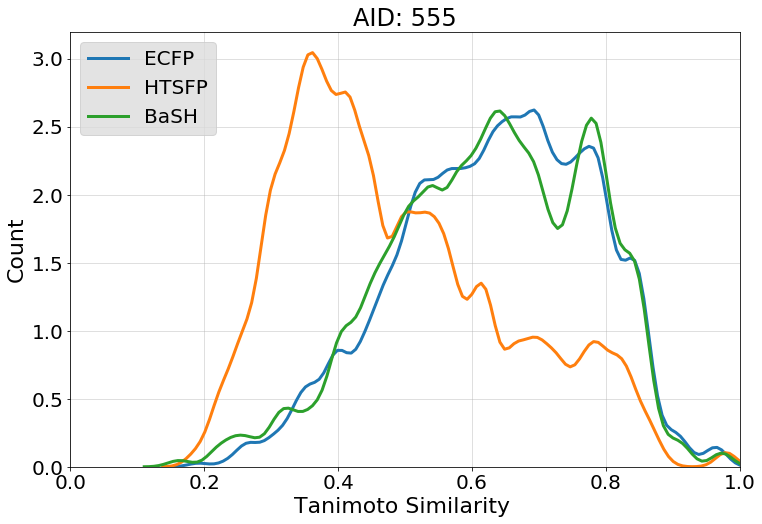

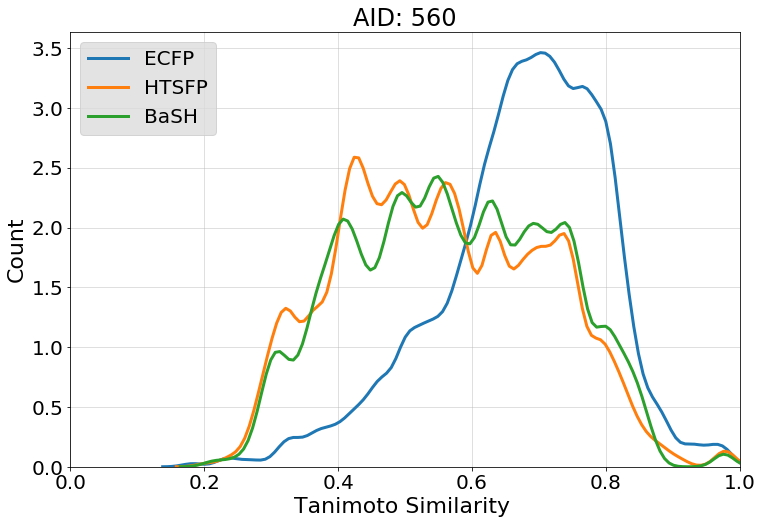

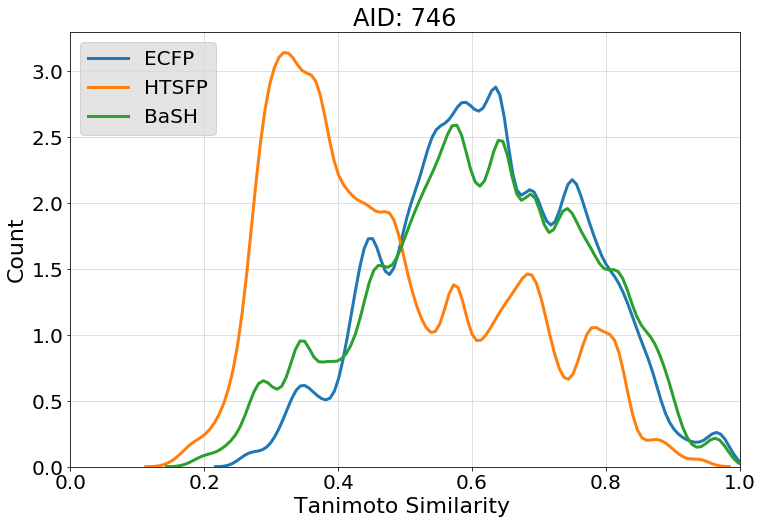

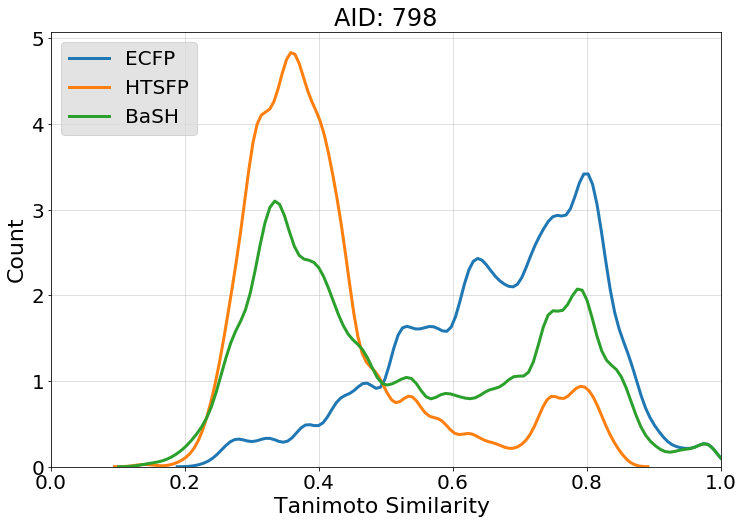

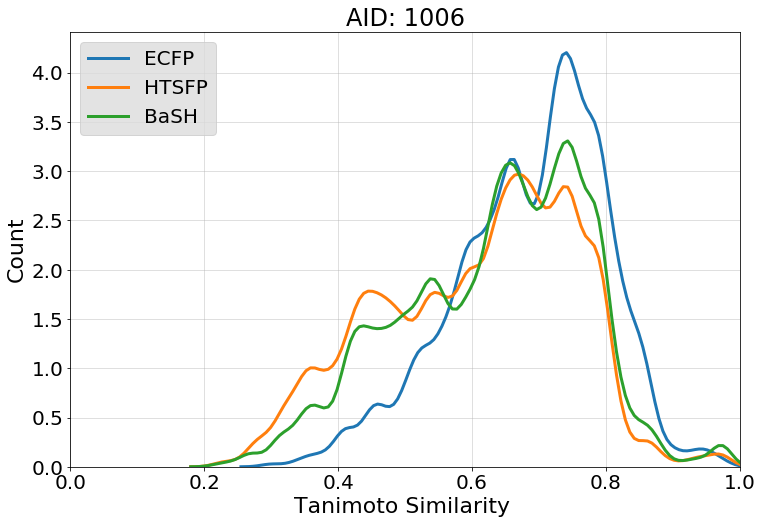

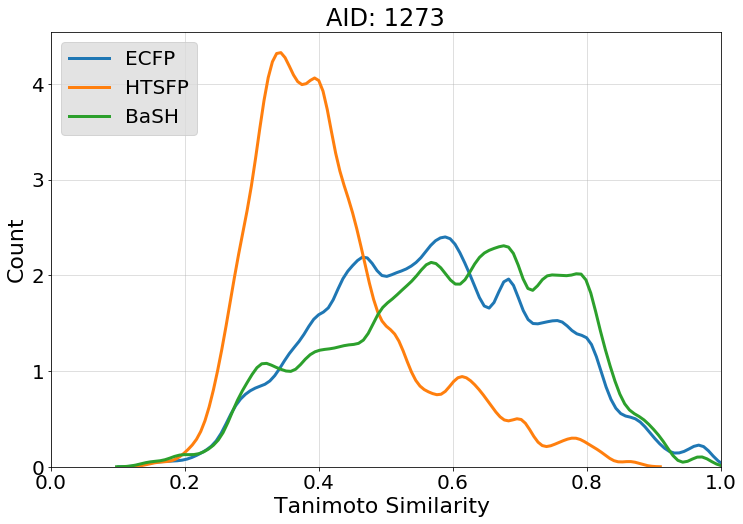

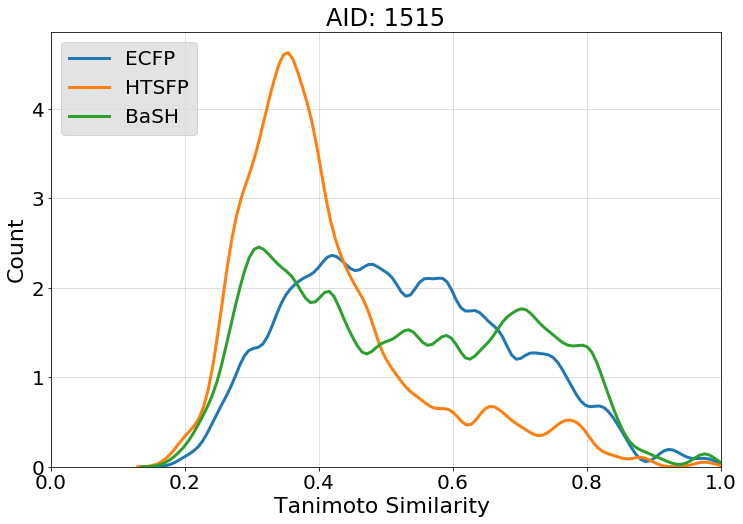

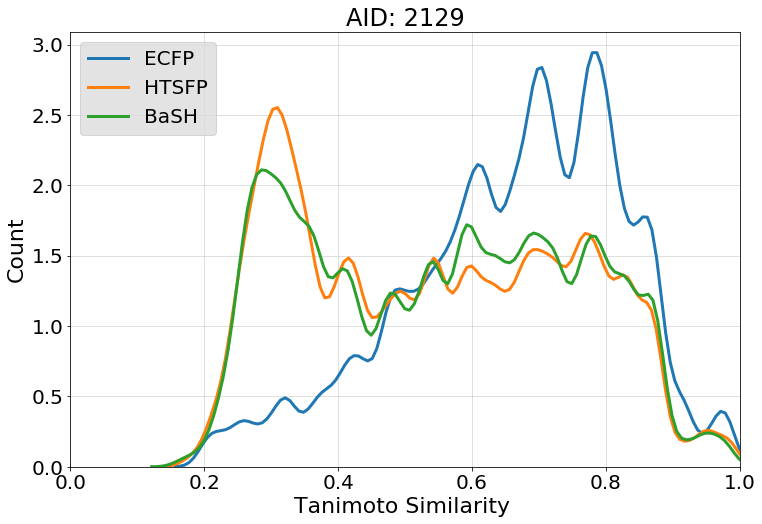

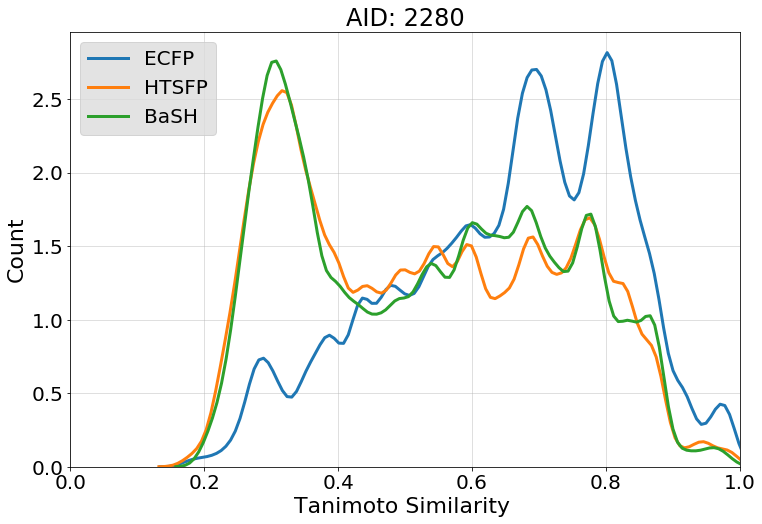

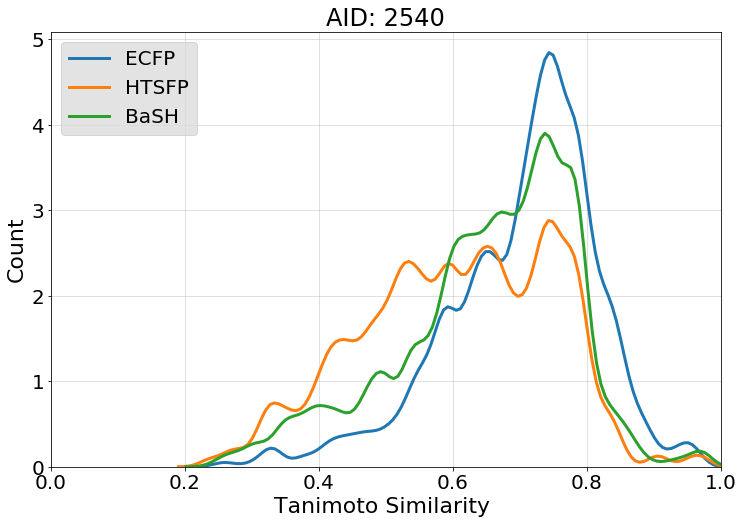

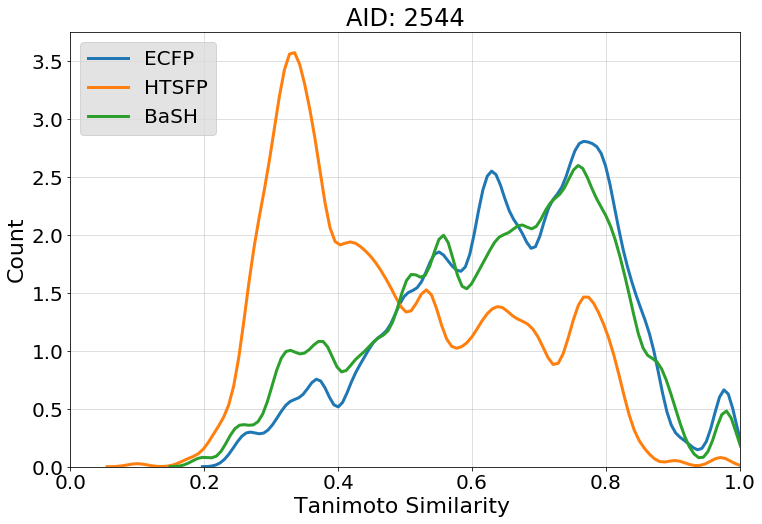

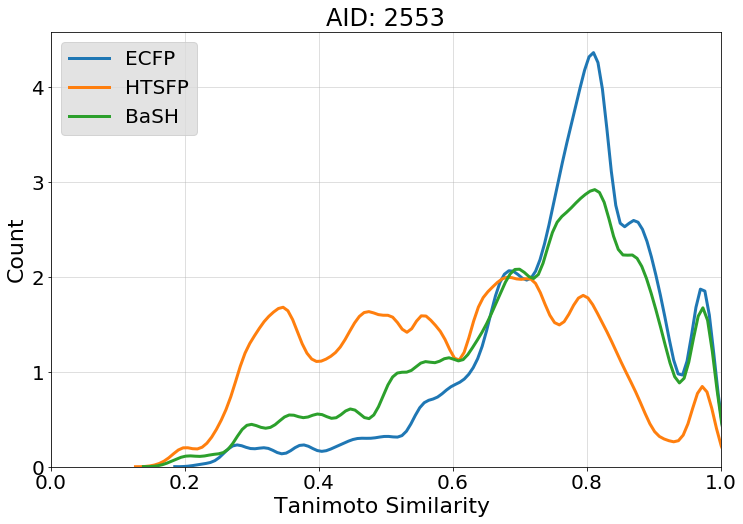

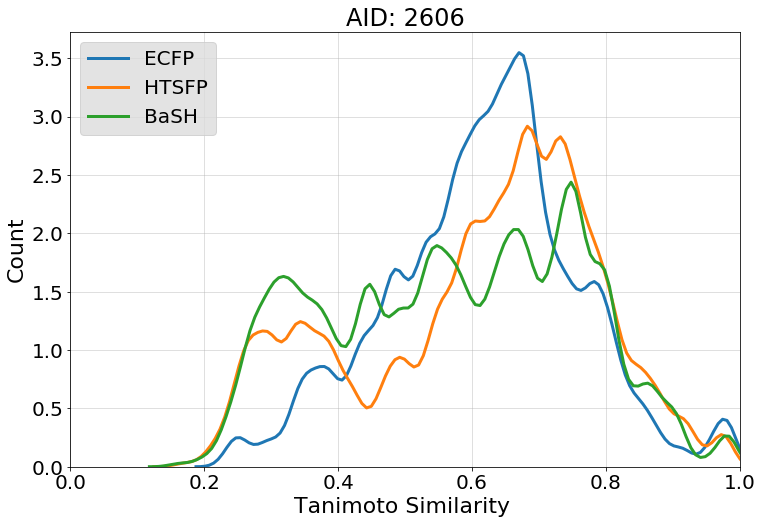

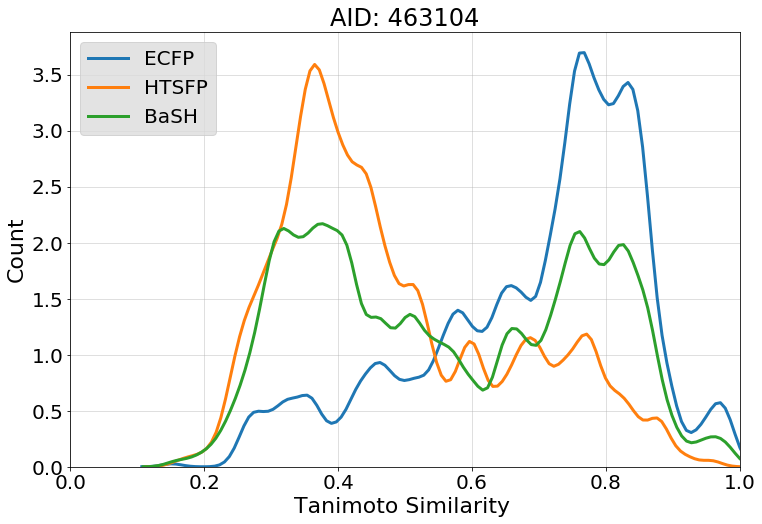

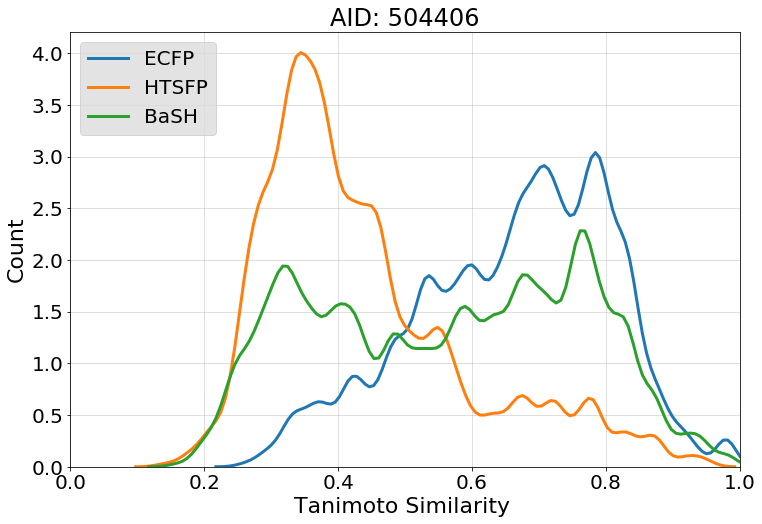

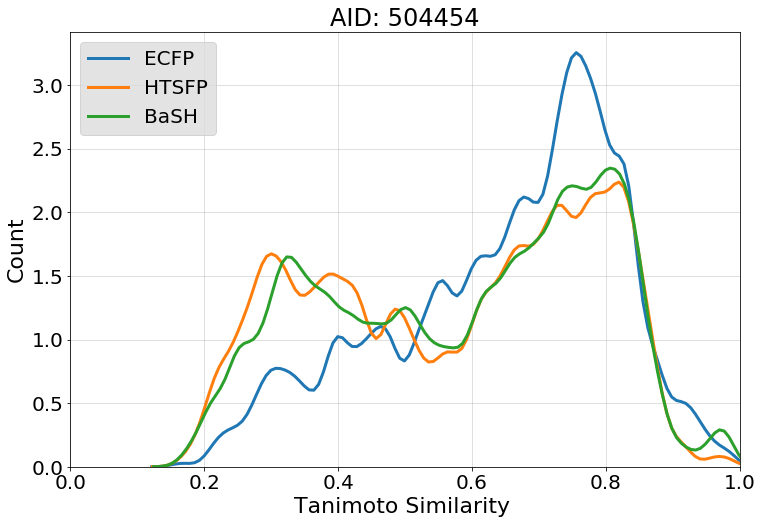

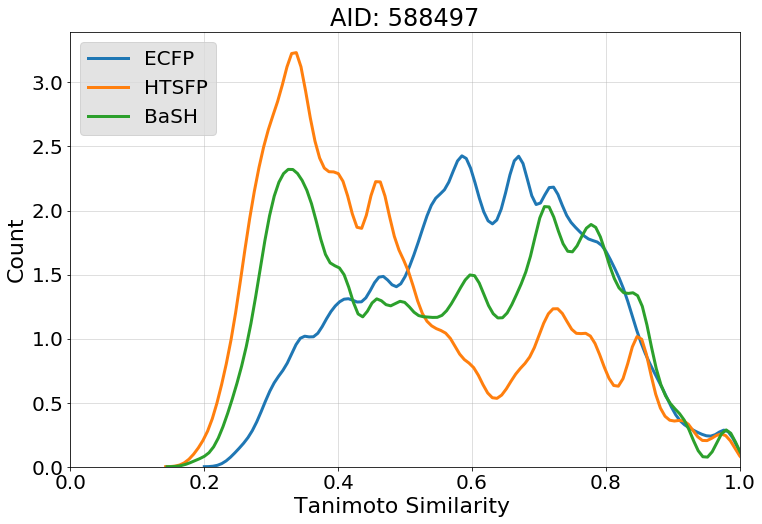

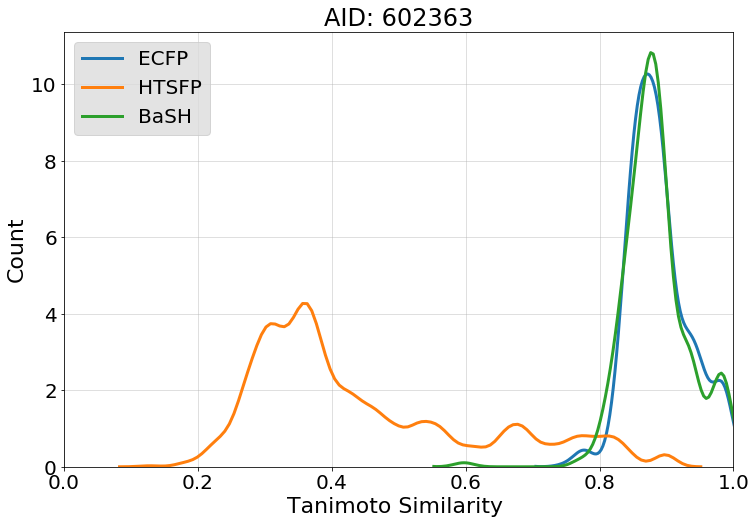

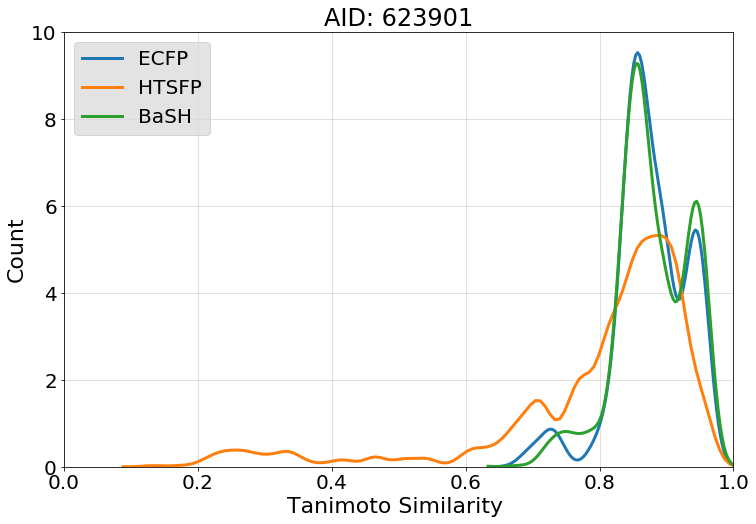

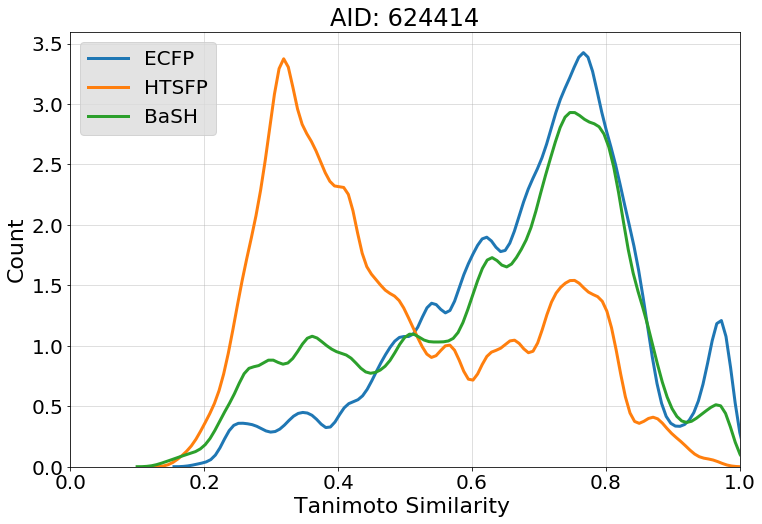

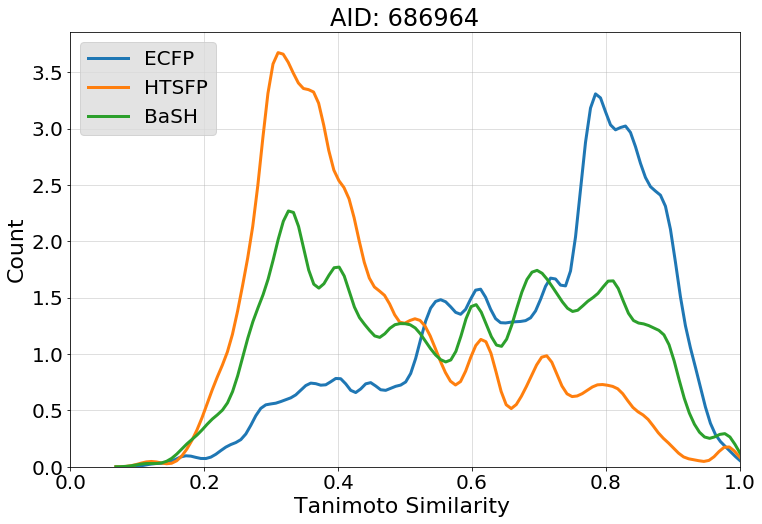

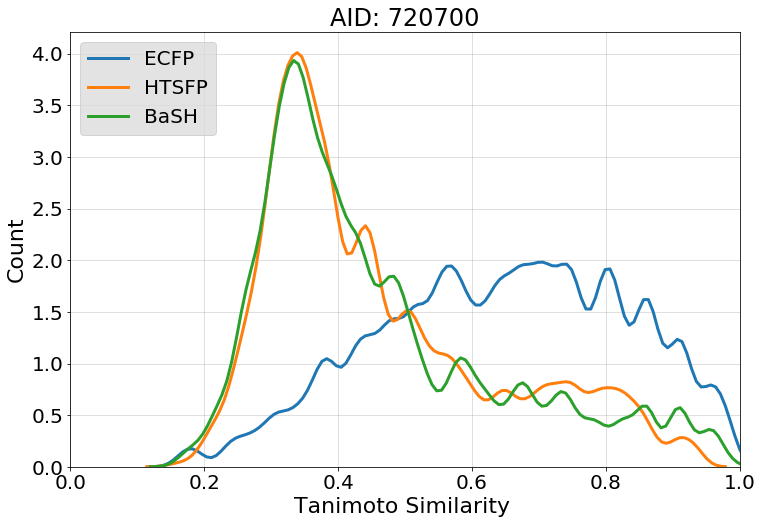


Figure S5. Compound diversity of top scoring 1000 compounds for each test assay. The nearest neighbor Tanimoto similarity was calculated for each of the 1000 compounds and plotted as a fitted histogram. The nearest neighbor similarity was calculated for each of the 3 predictive models BaSH: green, HTSFP: orange, and ECFP4: blue.


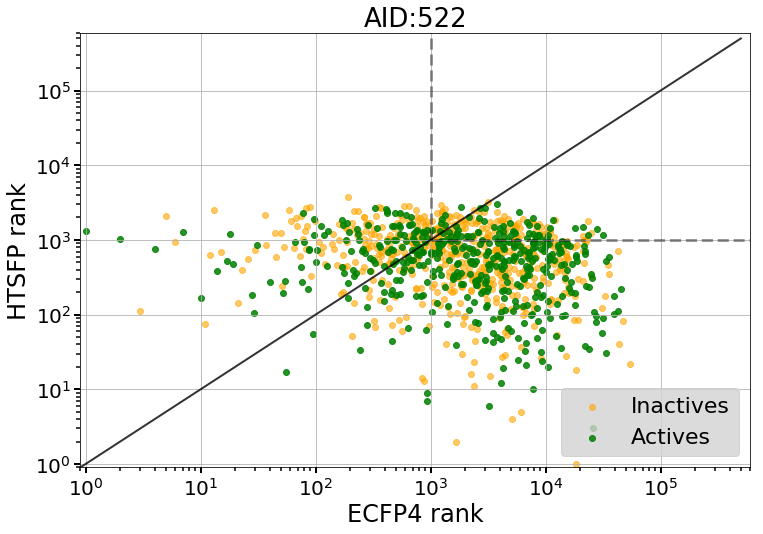

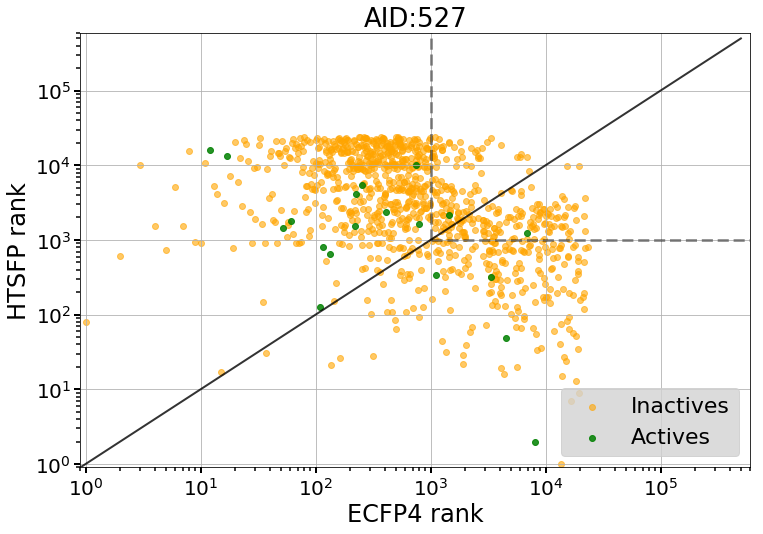

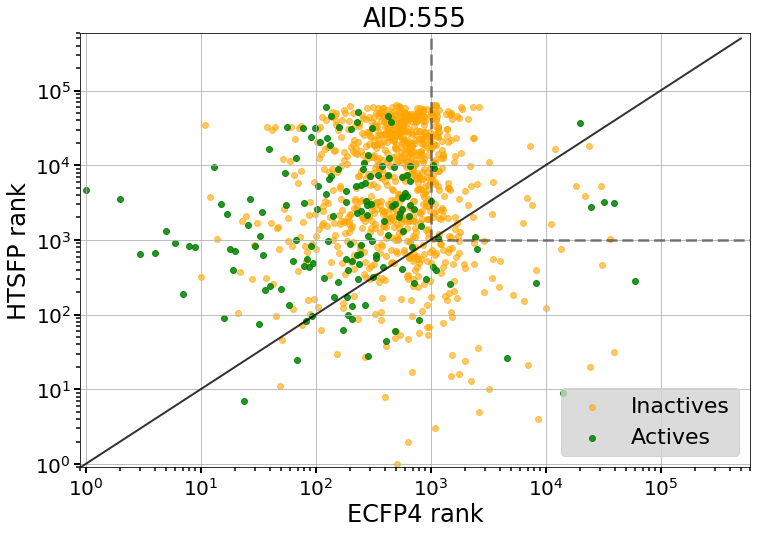

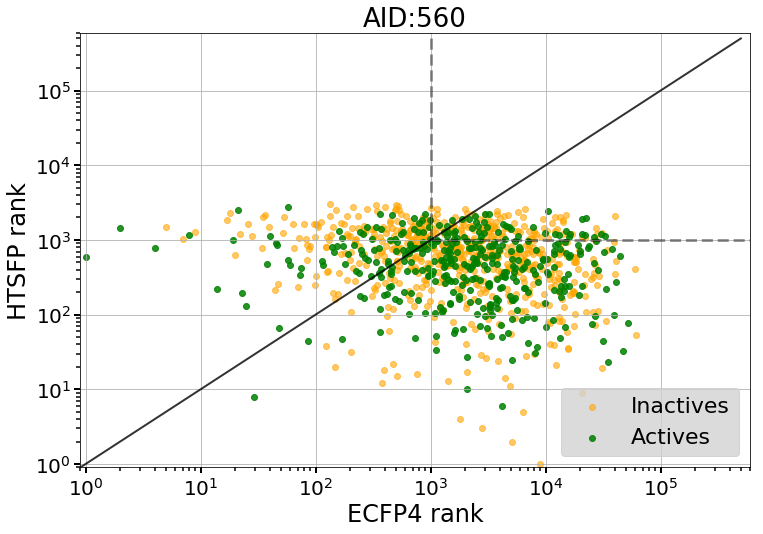

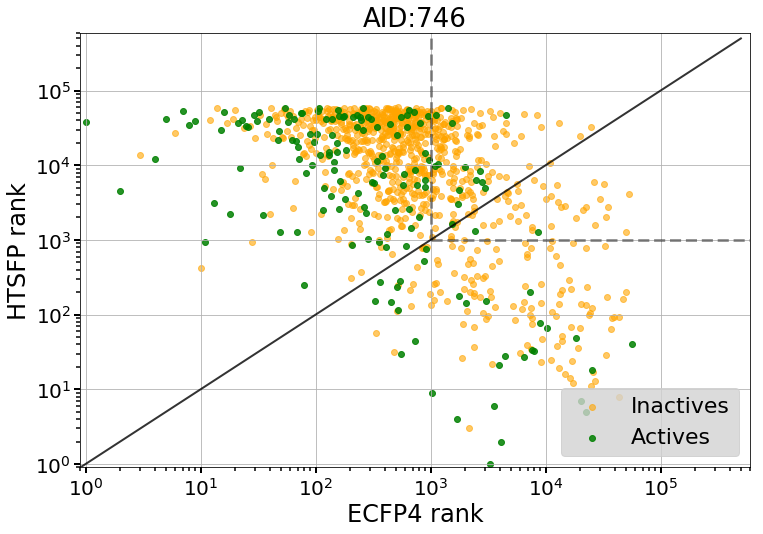

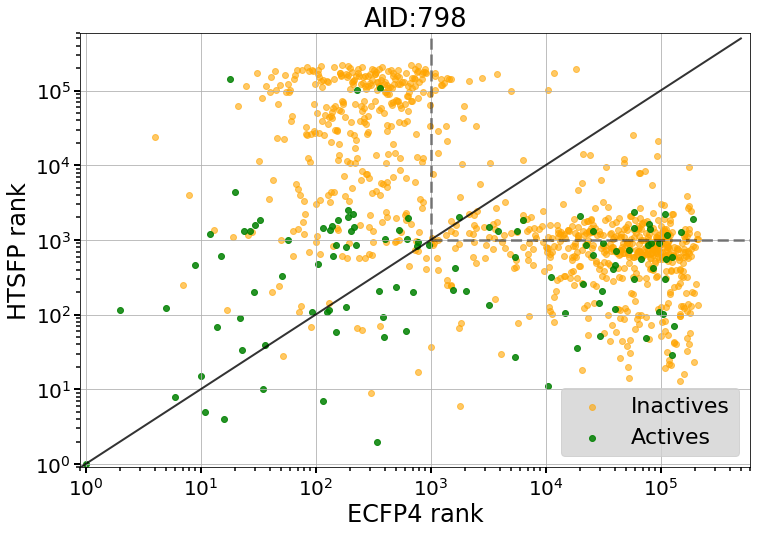

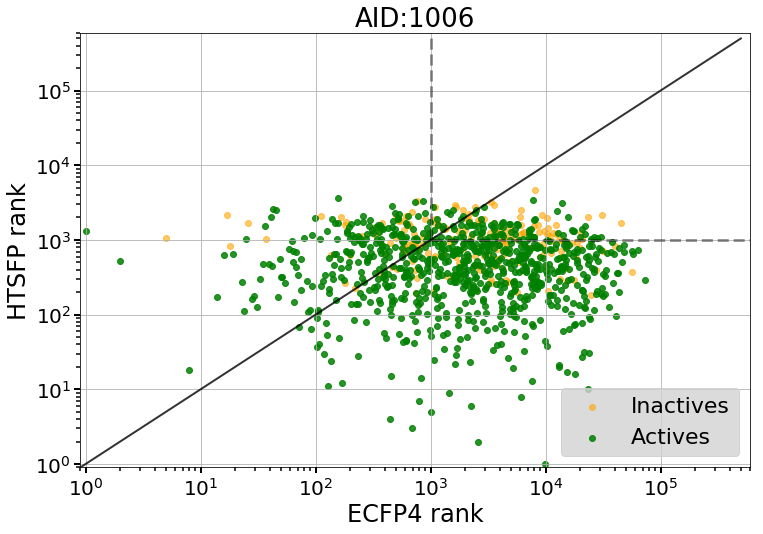

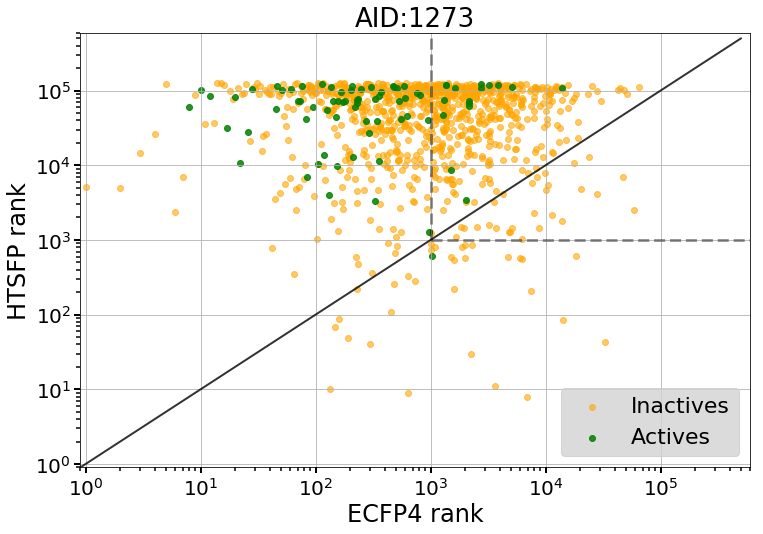

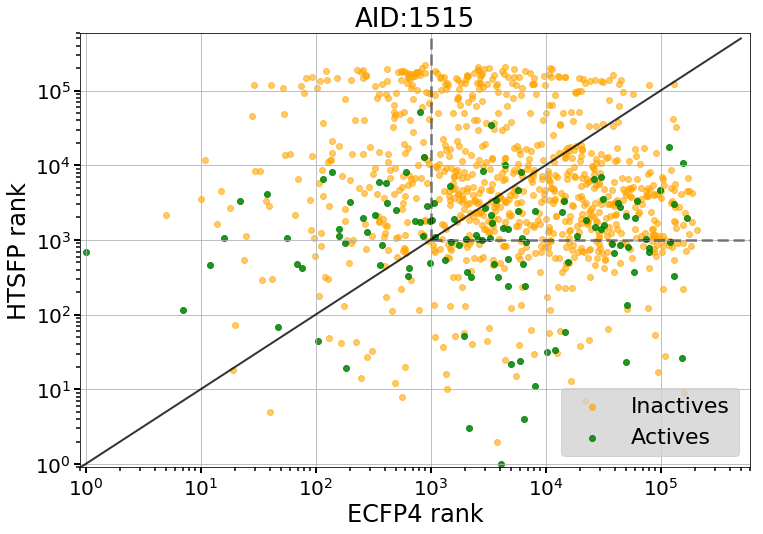

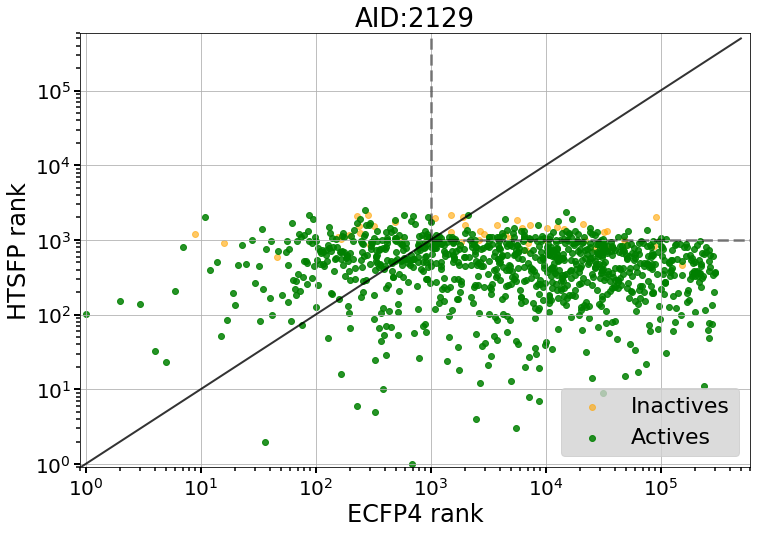

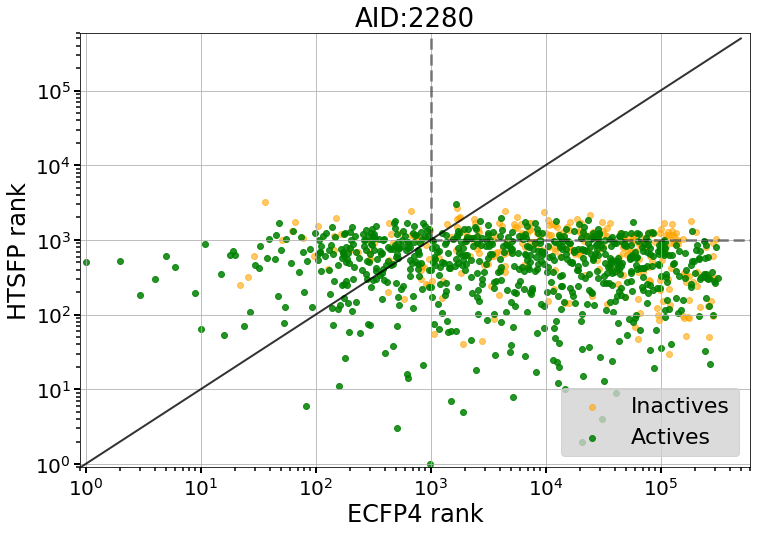

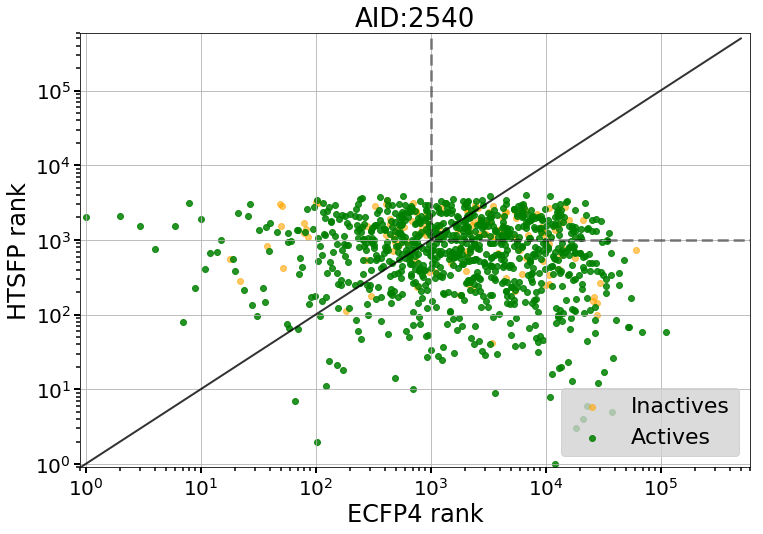

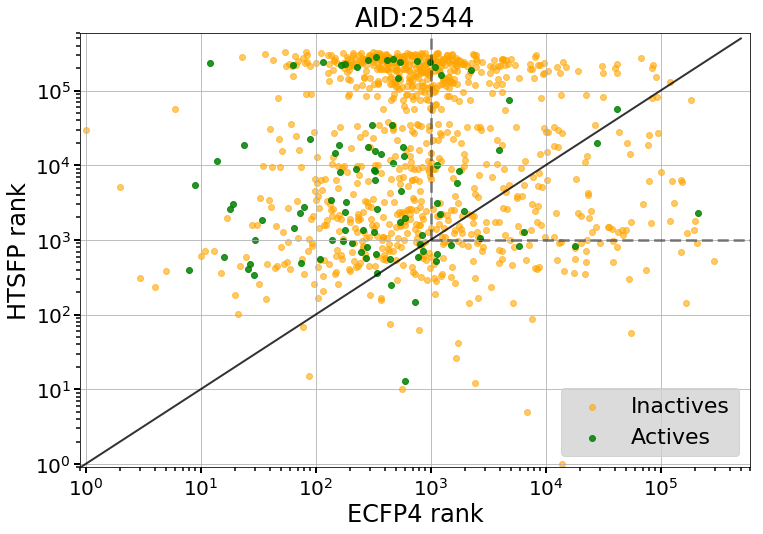

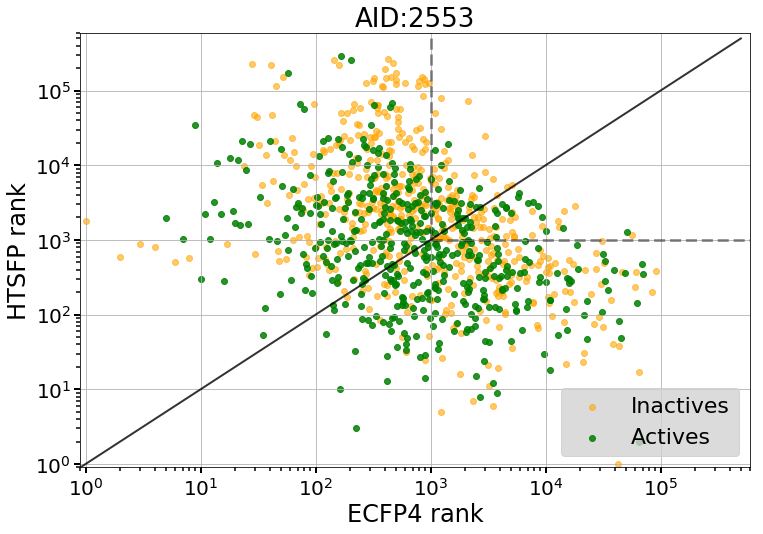

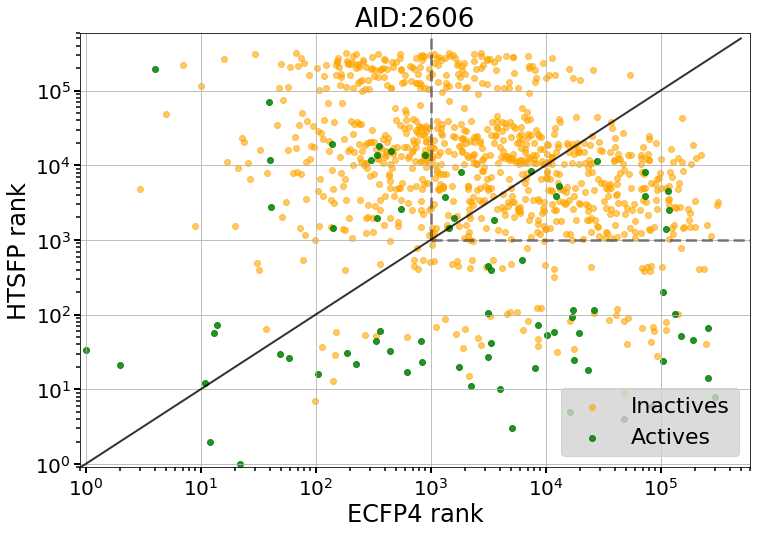

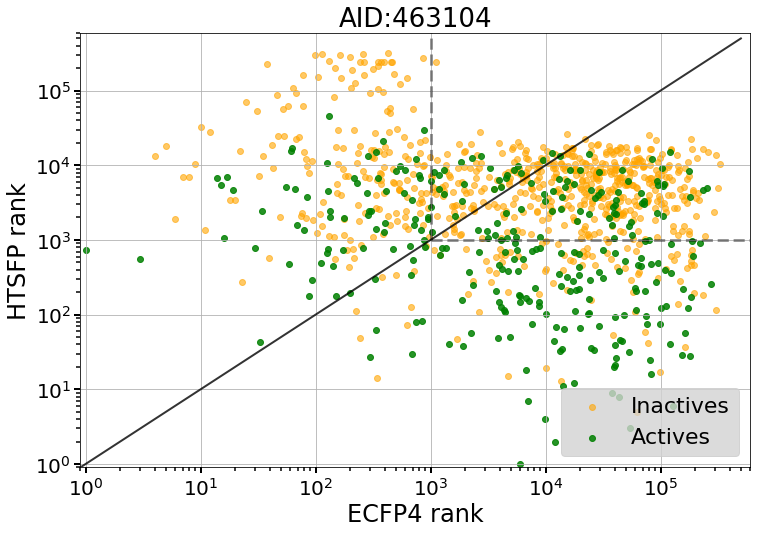

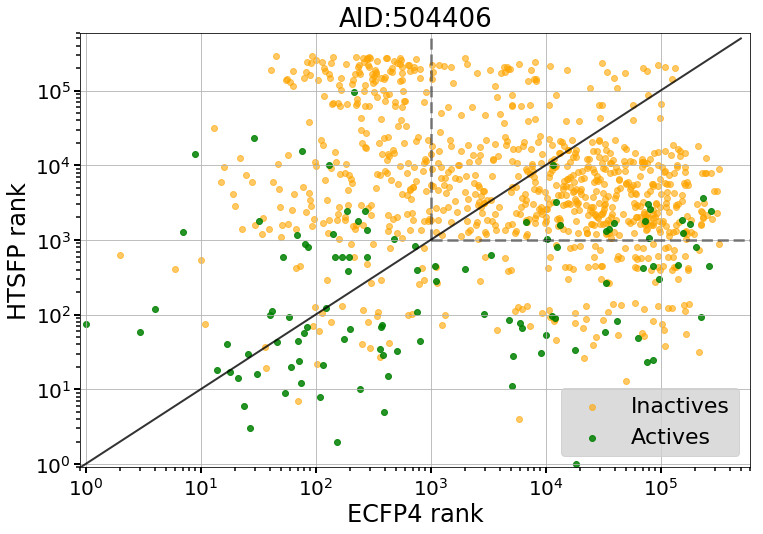

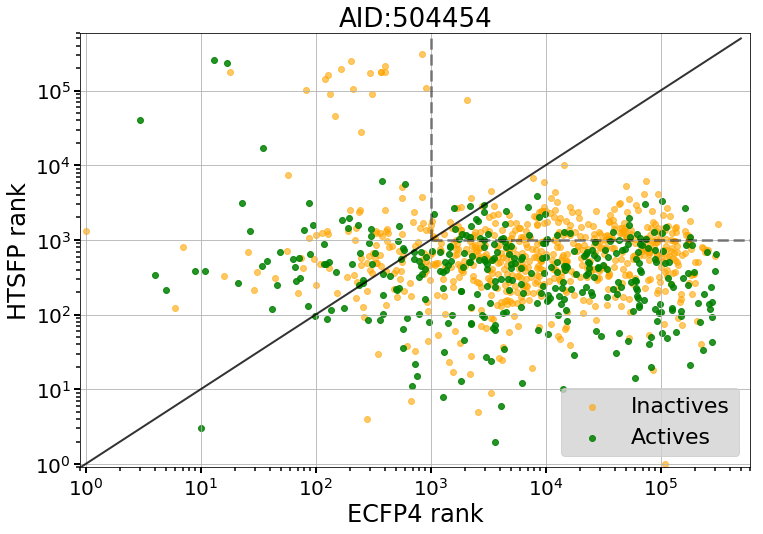

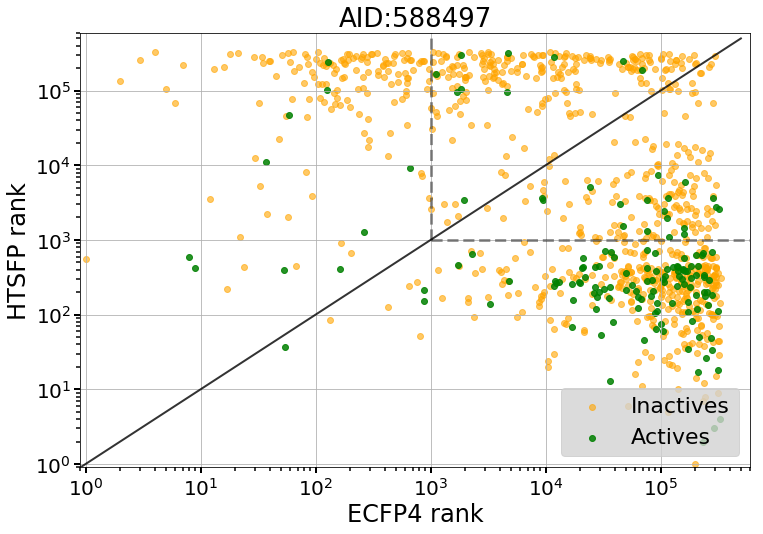

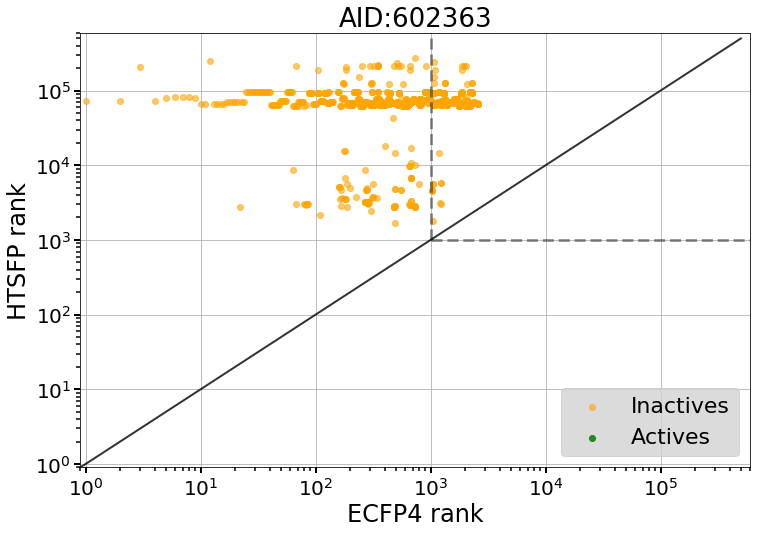


Figure S6. Comparison of compound rankings for the three prediction models. The top scoring 1000 compounds predicted using the BaSH are shown. The rankings of the same compounds in the HTSFP model (Y-axis) and the ECFP4 model (X-axis) are compared. The green and orange dots represent active and inactive compounds, respectively. The dashed line refers to rankings outside the top 1000 for both HTSFP and EFCP4. Plots A to H refer to each of the eight test assays.

Figure S7. Feature importance analysis of the combined fingerprint (BaSH). Features 0-560 correspond to the HTSFP portion (orange) and features 560-1584 correspond to the ECFP4 portion (blue) of the combined fingerprint. The light and dark shades of each feature refer to the mean and max values from the six-fold cross validation, respectively.

| **Assay ID** | **Index position** | **Feature Importance** | **Target** |
| --- | --- | --- | --- |
| 798 | Test Assay | | Coagulation factor XI |
| 800 | 532 | 0.044 | Coagulation factor XIIa light chain |
| 873 | 551 | 0.035 | Human kallikrein 5 (hK5) serine protease |
| 1046 | 17 | 0.02 | Prothrombin |
| 687 | 466 | 0.004 | Coagulation factor XI |
| 680 | 458 | 0.003 | Coagulation factor XI |
| 1515 | Test Assay | | Retinoblastoma Binding Protein 9 (RBBP9) |
| 1822 | 91 | 0.018 | M18 aspartyl aminopeptidase |
| 1022 | 10 | 0.015 | MCL-1 anti-apoptotic proteins |
| 720543 | 483 | 0.014 | Abhydrolase domain-containing protein 4 isoform 1 |
| 1053197 | 19 | 0.011 | Sialic acid acetylesterase (SIAE) |
| 504803 | 277 | 0.01 | HTRA serine peptidase 1 (HTRA1) |
| 2553 | Test Assay | | Transient Receptor Potential Cation Channel C6 (TRPC6) |
| 463111 | 198 | 0.02 | Regulator of G-protein signaling 4 isoform 2 |
| 588852 | 340 | 0.019 | Muscarinic acetylcholine receptor M1 |
| 488977 | 229 | 0.016 | High affinity choline transporter 1 |
| 743279 | 505 | 0.016 | Inhibitors of Inflammasome Signaling: IL-1-beta |
| 1063 | 20 | 0.014 | Inhibitors of Leishmania Parasite Growth |
| 463104 | Test Assay | | Activators of the adaptive arm of the Unfolded Protein response |
| 485346 | 216 | 0.074 | E3 ubiquitin-protein ligase Mdm2 isoform MDM2, protein Mdm4 |
| 2732 | 165 | 0.031 | Inhibitors Of CHOP To Regulate The unfolded protein response to ER stress |
| 449763 | 190 | 0.03 | Activators of the apoptotic arm of the unfolded Protein response |
| 588405 | 315 | 0.028 | Protein phosphatase 1 regulatory subunit 15A |
| 540308 | 293 | 0.014 | Melanocortin receptor 4 (MC4R) |

| **Assay ID** | **Index position** | **Feature Importance** | **Target** |
| --- | --- | --- | --- |
| 504454 | Test Assay | | Beta-2 adrenergic receptor |
| 588405 | 315 | 0.043 | Protein phosphatase 1 regulatory subunit 15A |
| 743279 | 505 | 0.026 | Inhibitors of Inflammasome Signaling: IL-1-beta |
| 488899 | 226 | 0.018 | MITF microphthalmia-associated transcription factor |
| 485346 | 217 | 0.016 | E3 ubiquitin-protein ligase Mdm2/MdmX |
| 624352 | 394 | 0.01 | Endothelial PAS domain-containing protein 1 |
| 588497 | Test Assay | | Botulinum neurotoxin light chain F protease |
| 588499 | 325 | 0.018 | Botulinum neurotoxin type A |
| 588501 | 326 | 0.016 | Lethal factor [Bacillus anthracis str. A2012] |
| 1962 | 105 | 0.005 | Likely tRNA 2'-phosphotransferase |
| 493008 | 236 | 0.003 | Troponin C, I, & T, and alpha tropomyosin |
| 624256 | 390 | 0.003 | Promoters of Myeloid Differentiation |
| 624414 | Test Assay | | Human Mucolipin Transient Receptor Potential 1 (TRPML1) |
| 588473 | 320 | 0.036 | Corticotropin-releasing hormone receptor 2 |
| 504660 | 269 | 0.027 | D(1A) dopamine receptor |
| 602248 | 351 | 0.021 | M1 muscarinic receptor(CHRM1) |
| 493084 | 241 | 0.019 | Thyrotropin-releasing hormone receptor |
| 2797 | 168 | 0.017 | Vasopressin V1a receptor |
| 686964 | Test Assay | | Methyl-CpG binding domain protein 2 |
| 687016 | 467 | 0.097 | E3 ubiquitin-protein ligase UHRF1 isoform 1 |
| 743269 | 504 | 0.022 | Integrase [Human immunodeficiency virus 1] |
| 588458 | 319 | 0.019 | DNA (cytosine-5)-methyltransferase 1 isoform b |
| 652257 | 457 | 0.015 | Protein arginine methyltransferase 1 (PRMT1) |
| 493091 | 243 | 0.014 | Carboxy-terminal domain RNA polymerase II polypeptide A small Phosphatase 1 isoform 1 |

Table S1. The top five most important features for each validation assay. Column 1 refers to PubChem assay ID number, column 2 refers to the position of the assay in the HTSFP portion of the fingerprint, and the ‘Imp_value’ refers to the relative importance value of the feature in the BaSH fingerprint.

| **FP type** | **Assay** | **ROC-AUC** | **MCC** | **Kappa** | **Precision** | **Recall** | **F1** | **A_count** | **N_count** | **TP** | **FN** | **FP** | **TN** |
| --- | --- | --- | --- | --- | --- | --- | --- | --- | --- | --- | --- | --- | --- |
| HTSFP | 522 | 0.970 | 0.442 | 0.344 | 0.226 | 0.931 | 0.364 | 1225 | 63682 | 1141 | 84 | 3899 | 59783 |
| ECFP | 522 | 0.849 | 0.207 | 0.196 | 0.165 | 0.312 | 0.216 | 1225 | 63682 | 382 | 843 | 1930 | 61752 |
| BaSH | 522 | 0.972 | 0.490 | 0.427 | 0.300 | 0.847 | 0.443 | 1225 | 63682 | 1037 | 188 | 2415 | 61267 |
| HTSFP | 527 | 0.689 | 0.029 | 0.024 | 0.018 | 0.063 | 0.028 | 64 | 24010 | 4 | 60 | 214 | 23796 |
| ECFP | 527 | 0.709 | 0.034 | 0.025 | 0.077 | 0.016 | 0.026 | 64 | 24010 | 1 | 63 | 12 | 23998 |
| BaSH | 527 | 0.775 | 0.082 | 0.054 | 0.222 | 0.031 | 0.055 | 64 | 24010 | 2 | 62 | 7 | 24003 |
| HTSFP | 555 | 0.796 | 0.127 | 0.065 | 0.039 | 0.497 | 0.073 | 316 | 64923 | 157 | 159 | 3819 | 61104 |
| ECFP | 555 | 0.786 | 0.310 | 0.309 | 0.336 | 0.291 | 0.312 | 316 | 64923 | 92 | 224 | 182 | 64741 |
| BaSH | 555 | 0.844 | 0.309 | 0.308 | 0.346 | 0.282 | 0.311 | 316 | 64923 | 89 | 227 | 168 | 64755 |
| HTSFP | 560 | 0.960 | 0.383 | 0.280 | 0.179 | 0.890 | 0.298 | 979 | 63928 | 871 | 108 | 3993 | 59935 |
| ECFP | 560 | 0.838 | 0.181 | 0.172 | 0.144 | 0.270 | 0.188 | 979 | 63928 | 264 | 715 | 1565 | 62363 |
| BaSH | 560 | 0.960 | 0.425 | 0.365 | 0.252 | 0.762 | 0.379 | 979 | 63928 | 746 | 233 | 2209 | 61719 |
| HTSFP | 746 | 0.664 | 0.071 | 0.048 | 0.034 | 0.216 | 0.058 | 366 | 59421 | 79 | 287 | 2277 | 57144 |
| ECFP | 746 | 0.865 | 0.238 | 0.230 | 0.313 | 0.186 | 0.233 | 366 | 59421 | 68 | 298 | 149 | 59272 |
| BaSH | 746 | 0.889 | 0.230 | 0.215 | 0.337 | 0.161 | 0.218 | 366 | 59421 | 59 | 307 | 116 | 59305 |
| HTSFP | 798 | 0.774 | 0.172 | 0.108 | 0.062 | 0.497 | 0.110 | 302 | 218414 | 150 | 152 | 2281 | 216133 |
| ECFP | 798 | 0.660 | 0.165 | 0.135 | 0.317 | 0.086 | 0.135 | 302 | 218414 | 26 | 276 | 56 | 218358 |
| BaSH | 798 | 0.795 | 0.289 | 0.241 | 0.540 | 0.156 | 0.242 | 302 | 218414 | 47 | 255 | 40 | 218374 |
| HTSFP | 1006 | 0.954 | 0.364 | 0.262 | 0.167 | 0.872 | 0.281 | 2976 | 192588 | 2595 | 381 | 12930 | 179658 |
| ECFP | 1006 | 0.914 | 0.291 | 0.247 | 0.174 | 0.547 | 0.264 | 2976 | 192588 | 1627 | 1349 | 7712 | 184876 |
| BaSH | 1006 | 0.970 | 0.519 | 0.484 | 0.366 | 0.765 | 0.495 | 2976 | 192588 | 2277 | 699 | 3952 | 188636 |
| HTSFP | 1273 | 0.496 | 0.007 | 0.004 | 0.011 | 0.084 | 0.020 | 1153 | 126144 | 97 | 1056 | 8376 | 117768 |
| ECFP | 1273 | 0.595 | 0.064 | 0.059 | 0.108 | 0.045 | 0.064 | 1153 | 126144 | 52 | 1101 | 430 | 125714 |
| BaSH | 1273 | 0.582 | 0.067 | 0.039 | 0.213 | 0.023 | 0.041 | 1153 | 126144 | 26 | 1127 | 96 | 126048 |
| HTSFP | 1515 | 0.679 | 0.092 | 0.073 | 0.047 | 0.196 | 0.076 | 445 | 217519 | 87 | 358 | 1770 | 215749 |
| ECFP | 1515 | 0.655 | 0.054 | 0.043 | 0.111 | 0.027 | 0.043 | 445 | 217519 | 12 | 433 | 96 | 217423 |
| BaSH | 1515 | 0.732 | 0.117 | 0.075 | 0.322 | 0.043 | 0.075 | 445 | 217519 | 19 | 426 | 40 | 217479 |
| HTSFP | 2129 | 0.936 | 0.534 | 0.495 | 0.364 | 0.796 | 0.500 | 2199 | 312803 | 1751 | 448 | 3053 | 309750 |
| ECFP | 2129 | 0.840 | 0.232 | 0.231 | 0.231 | 0.243 | 0.237 | 2199 | 312803 | 535 | 1664 | 1782 | 311021 |
| BaSH | 2129 | 0.947 | 0.688 | 0.685 | 0.634 | 0.751 | 0.688 | 2199 | 312803 | 1651 | 548 | 952 | 311851 |
| HTSFP | 2280 | 0.912 | 0.473 | 0.424 | 0.296 | 0.764 | 0.427 | 1419 | 323331 | 1084 | 335 | 2572 | 320759 |
| ECFP | 2280 | 0.813 | 0.224 | 0.219 | 0.284 | 0.181 | 0.221 | 1419 | 323331 | 257 | 1162 | 648 | 322683 |
| BaSH | 2280 | 0.933 | 0.622 | 0.616 | 0.544 | 0.715 | 0.618 | 1419 | 323331 | 1014 | 405 | 849 | 322482 |
| HTSFP | 2540 | 0.993 | 0.699 | 0.661 | 0.505 | 0.980 | 0.666 | 4119 | 326278 | 4036 | 83 | 3960 | 322318 |
| ECFP | 2540 | 0.920 | 0.255 | 0.187 | 0.122 | 0.611 | 0.204 | 4119 | 326278 | 2517 | 1602 | 18061 | 308217 |
| BaSH | 2540 | 0.994 | 0.754 | 0.736 | 0.608 | 0.944 | 0.740 | 4119 | 326278 | 3887 | 232 | 2506 | 323772 |
| HTSFP | 2544 | 0.592 | 0.060 | 0.049 | 0.032 | 0.122 | 0.050 | 393 | 330004 | 48 | 345 | 1467 | 328537 |
| ECFP | 2544 | 0.685 | 0.102 | 0.091 | 0.166 | 0.064 | 0.092 | 393 | 330004 | 25 | 368 | 126 | 329878 |
| BaSH | 2544 | 0.707 | 0.090 | 0.064 | 0.214 | 0.038 | 0.065 | 393 | 330004 | 15 | 378 | 55 | 329949 |
| HTSFP | 2553 | 0.805 | 0.205 | 0.143 | 0.093 | 0.538 | 0.158 | 3253 | 302361 | 1751 | 1502 | 17132 | 285229 |
| ECFP | 2553 | 0.803 | 0.240 | 0.240 | 0.232 | 0.267 | 0.248 | 3253 | 302361 | 869 | 2384 | 2873 | 299488 |
| BaSH | 2553 | 0.861 | 0.313 | 0.313 | 0.339 | 0.302 | 0.320 | 3253 | 302361 | 984 | 2269 | 1920 | 300441 |
| HTSFP | 2606 | 0.791 | 0.118 | 0.077 | 0.044 | 0.318 | 0.078 | 157 | 324594 | 50 | 107 | 1080 | 323514 |
| ECFP | 2606 | 0.747 | 0.156 | 0.129 | 0.295 | 0.083 | 0.129 | 157 | 324594 | 13 | 144 | 31 | 324563 |
| BaSH | 2606 | 0.862 | 0.288 | 0.244 | 0.521 | 0.159 | 0.244 | 157 | 324594 | 25 | 132 | 23 | 324571 |
| HTSFP | 463104 | 0.914 | 0.193 | 0.115 | 0.067 | 0.599 | 0.120 | 1100 | 330576 | 659 | 441 | 9192 | 321384 |
| ECFP | 463104 | 0.822 | 0.079 | 0.058 | 0.182 | 0.035 | 0.059 | 1100 | 330576 | 39 | 1061 | 175 | 330401 |
| BaSH | 463104 | 0.945 | 0.209 | 0.157 | 0.463 | 0.095 | 0.158 | 1100 | 330576 | 105 | 995 | 122 | 330454 |
| HTSFP | 504406 | 0.868 | 0.179 | 0.107 | 0.060 | 0.541 | 0.108 | 194 | 323720 | 105 | 89 | 1643 | 322077 |
| ECFP | 504406 | 0.792 | 0.209 | 0.175 | 0.386 | 0.113 | 0.175 | 194 | 323720 | 22 | 172 | 35 | 323685 |
| BaSH | 504406 | 0.884 | 0.373 | 0.346 | 0.551 | 0.253 | 0.346 | 194 | 323720 | 49 | 145 | 40 | 323680 |
| HTSFP | 504454 | 0.776 | 0.217 | 0.186 | 0.126 | 0.395 | 0.191 | 1446 | 337839 | 571 | 875 | 3967 | 333872 |
| ECFP | 504454 | 0.699 | 0.102 | 0.086 | 0.188 | 0.057 | 0.088 | 1446 | 337839 | 83 | 1363 | 358 | 337481 |
| BaSH | 504454 | 0.838 | 0.284 | 0.282 | 0.316 | 0.259 | 0.285 | 1446 | 337839 | 375 | 1071 | 810 | 337029 |
| HTSFP | 588497 | 0.605 | 0.081 | 0.058 | 0.036 | 0.206 | 0.061 | 780 | 339542 | 161 | 619 | 4300 | 335242 |
| ECFP | 588497 | 0.605 | 0.026 | 0.022 | 0.047 | 0.015 | 0.023 | 780 | 339542 | 12 | 768 | 245 | 339297 |
| BaSH | 588497 | 0.658 | 0.145 | 0.115 | 0.295 | 0.072 | 0.115 | 780 | 339542 | 56 | 724 | 134 | 339408 |
| HTSFP | 602363 | 0.597 | 0.035 | 0.014 | 0.008 | 0.200 | 0.016 | 446 | 346711 | 89 | 357 | 10501 | 336210 |
| ECFP | 602363 | 0.515 | -0.006 | -0.002 | 0.000 | 0.009 | 0.001 | 446 | 346711 | 4 | 442 | 14101 | 332610 |
| BaSH | 602363 | 0.575 | -0.006 | -0.002 | 0.000 | 0.009 | 0.001 | 446 | 346711 | 4 | 442 | 14430 | 332281 |
| HTSFP | 623901 | 0.510 | 0.017 | 0.007 | 0.005 | 0.094 | 0.010 | 470 | 332289 | 44 | 426 | 8289 | 324000 |
| ECFP | 623901 | 0.641 | 0.025 | 0.009 | 0.006 | 0.162 | 0.012 | 470 | 332289 | 76 | 394 | 12058 | 320231 |
| BaSH | 623901 | 0.745 | 0.021 | 0.007 | 0.005 | 0.147 | 0.010 | 470 | 332289 | 69 | 401 | 13087 | 319202 |
| HTSFP | 624414 | 0.791 | 0.281 | 0.229 | 0.146 | 0.546 | 0.231 | 482 | 399857 | 263 | 219 | 1534 | 398323 |
| ECFP | 624414 | 0.828 | 0.173 | 0.171 | 0.203 | 0.149 | 0.172 | 482 | 399857 | 72 | 410 | 283 | 399574 |
| BaSH | 624414 | 0.907 | 0.441 | 0.441 | 0.429 | 0.454 | 0.442 | 482 | 399857 | 219 | 263 | 291 | 399566 |
| HTSFP | 686964 | 0.786 | 0.282 | 0.237 | 0.156 | 0.522 | 0.240 | 1149 | 368790 | 600 | 549 | 3245 | 365545 |
| ECFP | 686964 | 0.637 | 0.105 | 0.101 | 0.145 | 0.079 | 0.103 | 1149 | 368790 | 91 | 1058 | 535 | 368255 |
| BaSH | 686964 | 0.789 | 0.380 | 0.369 | 0.487 | 0.299 | 0.371 | 1149 | 368790 | 344 | 805 | 362 | 368428 |
| HTSFP | 720700 | 0.633 | 0.117 | 0.098 | 0.071 | 0.237 | 0.110 | 3123 | 366816 | 740 | 2383 | 9647 | 357169 |
| ECFP | 720700 | 0.577 | 0.017 | 0.016 | 0.033 | 0.015 | 0.021 | 3123 | 366816 | 48 | 3075 | 1385 | 365431 |
| BaSH | 720700 | 0.658 | 0.143 | 0.123 | 0.259 | 0.084 | 0.127 | 3123 | 366816 | 262 | 2861 | 750 | 366066 |

Table 2. Overview of all metrics and confusion matrix for all descriptor types and assays.

Figure S8. Example chemical structures from assay 1515 that were only correctly predicted by the BaSH fingerprint. The figure shows six compounds which indicate the structural diversity of compounds correctly predicted using the BaSH fingerprint.
